# Curcumin-Based Nanoformulations for Oral Health: Mechanistic Insights, Antimicrobial Efficacy, and Future Clinical Perspectives

**DOI:** 10.3390/biomedicines14040815

**Published:** 2026-04-02

**Authors:** Dana-Emanuela Pitic (Coţ), Ramona-Amina Popovici, Codruţa-Eliza Ille, Ioana-Cristina Talpoş-Niculescu, Adelina Chevereşan, Daniel Pop, Alexandra-Ioana Dănilă, Emilia Daliana Muntean, Iasmina Denisa Boantă, Andreea Kis, Ciprian Stroia

**Affiliations:** 1 Faculty of Dental Medicine, “Victor Babes” University of Medicine and Pharmacy, Revolutiei Ave. 1989, No. 9, 300580 Timisoara, Romaniaramona.popovici@umft.ro (R.-A.P.); ille.codruta@umft.ro (C.-E.I.); pop.daniel@umft.ro (D.P.); kis.andreea@umft.ro (A.K.); 2 Doctoral School, Faculty of Dental Medicine, “Victor Babes” University of Medicine and Pharmacy, Revolutiei Ave. 1989, No. 9, 300580 Timisoara, Romania; 3 Research Center of Digital and Advanced Technique for Endodontic, Restorative and Prosthetic Treatment (TADERP), “Victor Babes” University of Medicine and Pharmacy, Revolutiei Ave. 1989, No. 9, 300580 Timisoara, Romania; 4 Faculty of Medicine, “Victor Babes” University of Medicine and Pharmacy, 2nd Eftimie Murgu Square, 300041 Timisoara, Romania; alexandra.danila@umft.ro (A.-I.D.); emilia.muntean@umft.ro (E.D.M.); iasmina.boanta@rezident.umft.ro (I.D.B.); 5 Faculty of Agriculture, University of Life Sciences “King Mihai I” from Timisoara, 119 Calea Aradului, 300645 Timisoara, Romania; ciprian.stroia@usvt.ro

**Keywords:** *Curcuma longa* L., curcumin nanoformulations, oral health, biofilm inhibition, antioxidant mechanism, drug-delivery systems, nanoparticles, periodontal disease

## Abstract

**Background/Objectives**: Oral diseases remain among the most prevalent noncommunicable conditions worldwide, with biofilm-driven dysbiosis playing a central role in dental caries, gingivitis, periodontitis, and oral candidiasis. Curcumin has attracted considerable interest because of its anti-inflammatory, antioxidant, antimicrobial, and regenerative properties. However, its clinical use remains limited by poor water solubility, chemical instability, rapid metabolism, and low bioavailability. This review aimed to provide a comprehensive analysis of curcumin-based nanoformulations for oral health applications, with emphasis on their mechanistic actions, antibiofilm activity, and translational relevance. **Methods**: This review examined representative nanocarrier systems developed for curcumin delivery in oral health. These included polymeric nanoparticles, nanomicelles and nanoemulsions, solid lipid nanoparticles and nanostructured lipid carriers, nanogels, hydrogels, mucoadhesive films, and metallic or hybrid nanosystems. The analysis focused on molecular mechanisms of action, antimicrobial and antibiofilm effects against major oral pathogens, and key translational challenges. **Results/Findings:** Across the reviewed studies, nanoformulations consistently improved curcumin solubility, stability, tissue penetration, mucosal retention, and controlled release. Mechanistically, they enhanced anti-inflammatory activity through inhibition of nuclear factor kappa B (NF-κB), strengthened antioxidant defenses via the nuclear factor erythroid 2-related factor 2/heme oxygenase-1 (Nrf2/HO-1) axis, supported tissue repair and osteogenic responses, disrupted oral biofilms, and modulated local immune responses. Antimicrobial activity was reported against *Streptococcus mutans*, *Porphyromonas gingivalis*, *Aggregatibacter actinomycetemcomitans*, and *Candida albicans*, with reduced exopolysaccharide production, impaired adhesion, and improved biofilm penetration. **Conclusions**: Curcumin-based nanoformulations represent promising adjunctive platforms for oral healthcare. However, their clinical translation still requires improved stability in the oral-environment standardized manufacturing and characterization, rigorous safety evaluation, and well-designed controlled clinical studies.

## 1. Introduction

**Figure 1:**
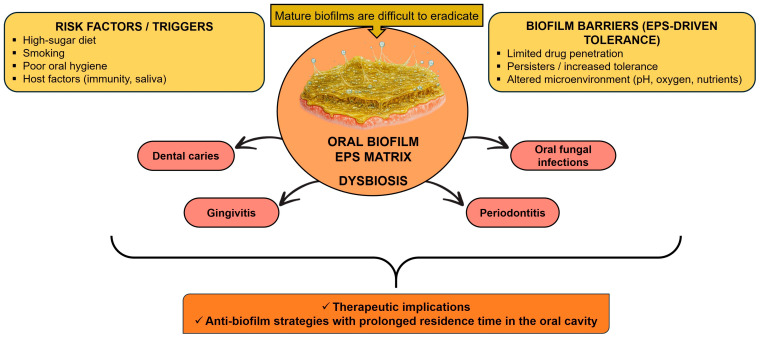
Schematic representation of oral biofilm dysbiosis, associated oral diseases, and major therapeutic challenges. Risk factors such as a high-sugar diet, smoking, poor oral hygiene, and host-related factors promote the development of dysbiotic oral biofilms. Once established, mature biofilms exhibit extracellular polymeric substance (EPS)-driven tolerance, limited drug penetration, increased persistence, and microenvironmental alterations, thereby contributing to dental caries, gingivitis, periodontitis, and oral fungal infections. These features highlight the therapeutic need for anti-biofilm strategies with prolonged residence time in the oral cavity.

Globally, oral diseases rank among the most prevalent health problems and impose a substantial burden on quality of life. According to recent estimates from the World Health Organization (WHO), dental caries, gingivitis, periodontal diseases, and oral fungal infections remain among the most widespread noncommunicable conditions worldwide [1,2,3]. Oral conditions affect approximately 3.5 billion people, underscoring the scale of the problem across diverse populations and settings [4,5]. Their etiology is multifactorial and involves interaction among the oral microbiome, the host immune response, environmental exposure, and individual behaviors such as unhealthy diet, smoking, and inadequate oral hygiene [2,6,7,8,9,10,11,12]. Within this framework, oral biofilm dysbiosis plays a central pathogenic role. Oral biofilms provide a dynamic and resilient microbial environment in which cariogenic and periodontopathogenic processes develop and persist, while the extracellular matrix limits the effectiveness of conventional antimicrobial therapies [13,14,15,16]. Ecological shifts within dental plaque promote acidogenic and acid-tolerant microbial communities, thereby contributing to caries development and increasing the broader risk of periodontal disease [14,16,17,18,19,20]. The pathogenic role of oral biofilm dysbiosis, its major clinical consequences, and the need for prolonged anti-biofilm therapeutic strategies are schematically illustrated in Figure 1.

Conventional therapeutic approaches in dentistry rely mainly on antiseptics (e.g., chlorhexidine (CHX)), fluorides, antibiotics, and anti-inflammatory agents. Although these interventions remain clinically important, their effectiveness may be limited by mature biofilm resistance, rapid clearance from the oral cavity, short mucosal contact time, and the potential for adverse effects or reduced efficacy in complex microbial communities [14,15,16,21,22,23,24,25,26]. Therefore, these limitations have stimulated researchers’ interest in developing alternative, biocompatible therapies that are effective against biofilms, exhibit prolonged residence time, and have a reduced potential to induce resistance [22,23,27,28,29,30,31,32].

Curcumin, a polyphenol derived from the rhizomes of *Curcuma longa* (turmeric), has attracted considerable interest in biomedical research because of its broad pharmacological profile, including anti-inflammatory, antioxidant, antibacterial, antifungal, anticancer, and neuroprotective effects. Experimental studies have shown that curcumin modulates key pathways involved in inflammation and oxidative stress, including suppression of nuclear factor kappa B (NF-κB) signaling, downregulation of cyclooxygenase-2 (COX-2), attenuation of inducible nitric oxide synthase (iNOS) expression, activation of the nuclear factor erythroid 2-related factor 2/heme oxygenase-1 (Nrf2/HO-1) axis, and inhibition of oral pathogens such as *Streptococcus mutans*, *Porphyromonas gingivalis*, and *Candida albicans* [33,34,35,36,37]. In addition, curcumin is generally considered safe at therapeutic concentrations. However, its clinical translation remains limited by unfavorable physicochemical and pharmacokinetic properties, including high hydrophobicity, extremely low water solubility, instability at neutral or alkaline pH, poor intestinal absorption, light sensitivity, rapid metabolism, and systemic elimination [33,34,38,39]. Collectively, these factors result in low oral bioavailability and limited tissue exposure after administration of native curcumin. Clinical and pharmacokinetic studies indicate that biologically active concentrations are difficult to achieve with conventional oral formulations, often requiring very high doses or alternative delivery strategies [34,35,40,41]. Accordingly, improving curcumin solubility, stability, and retention has become a major objective in formulation research. In this context, nanotechnology offers promising opportunities to protect curcumin from degradation, enhance its bioavailability, and improve its therapeutic performance in oral and systemic applications [33,34,38,42,43,44].

Curcumin-based nanoformulations have been designed to address the challenges associated with curcumin’s hydrophobicity, degradation, and bioavailability. These strategies aim to improve solubility and stability in aqueous media, protect curcumin against premature enzymatic and chemical degradation, enhance intestinal absorption and systemic exposure, and facilitate penetration into microbial biofilms [45,46,47]. In addition, nanocarrier platforms such as polymeric nanoparticles, nanomicelles, solid lipid nanoparticles, or mucoadhesive hydrogels can be engineered for controlled release, local targeting, and synergistic antimicrobial activity. All of these features are particularly relevant in the oral cavity, where dynamic conditions and the presence of biofilms impose substantial demands on effective therapy. For example, micellar systems and various curcumin-based nanoformulations may be considered ideal vehicles for significantly improving curcumin dispersibility and solubility in aqueous media, to levels substantially greater than those of the native compound, as well as for enhancing its stability in the gastrointestinal environment [46,47,48,49]. In addition, organogel-based nanoemulsions have been specifically developed to increase oral bioavailability by improving solubility and facilitating dispersion in the intestinal environment, thereby enhancing curcumin solubility and absorption [49]. Beyond solubility, protection against chemical and enzymatic degradation in the gastrointestinal tract is essential for achieving meaningful systemic exposure after oral administration. To mitigate curcumin degradation, hydrophobic microenvironments and nanoencapsulation strategies have been proposed, providing a protective matrix and controlled release [39,50]. Moreover, encapsulation of curcumin in various nanoformulations results in improved stability and protection against degradation; therefore, nanoencapsulation may attenuate rapid metabolism and chemical instability compared with free curcumin [48,51]. Interactions with the biological environment (e.g., plasma proteins) are also important for in vivo persistence and distribution after oral administration of curcumin [52]. Understanding these interactions further strengthens the rationale for using nanoformulations to stabilize curcumin before absorption and systemic distribution [52].

Another major advantage of curcumin-based nanoformulations is their potential to achieve controlled or sustained release, thereby supporting prolonged presence in the gastrointestinal tract or controlled systemic exposure. Different curcumin-based nanosystems with sustained or delayed-release characteristics have been formulated to ensure a more gradual release of curcumin over time. These delivery nanosystems have shown improved therapeutic profiles and a reduced frequency of administration for oral use [46,47,53].

**Figure 2:**
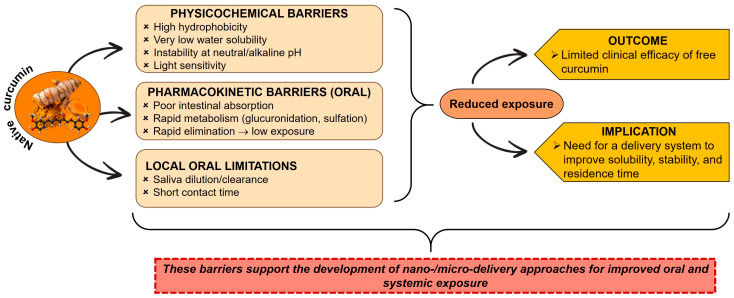
Schematic overview of the physicochemical, pharmacokinetic, and local oral barriers limiting the clinical efficacy of native curcumin. Native curcumin is affected by high hydrophobicity, very low water solubility, instability at neutral or alkaline pH, and light sensitivity, as well as poor intestinal absorption, rapid metabolism, and rapid elimination. In the oral cavity, salivary dilution, clearance, and short contact time further reduce local exposure. Collectively, these barriers result in limited oral and systemic bioavailability and support the need for delivery systems capable of improving solubility, stability, and residence time.

Therefore, improvements in solubility, stability, and release control consistently represent prerequisites for the efficacy of orally administered curcumin, and nanoformulations collectively illustrate multiple complementary approaches for achieving the desired pharmacokinetic profile, improved therapeutic potential, and adequate tissue exposure after oral administration [45,46,47,48,49,52,53,54,55]. The major physicochemical, pharmacokinetic, and local oral barriers limiting the clinical efficacy of native curcumin are summarized in Figure 2.

In the context of the considerations presented above, and given the growing interest in nanoformulations for curcumin delivery, this review aims to provide a comprehensive and clinically relevant overview of curcumin-based nanoformulations for oral health applications. Specifically, the objectives of this review are to: (i) describe the principal classes of nanocarriers investigated for curcumin delivery in the oral environment; (ii) summarize the molecular mechanisms through which these systems exert anti-inflammatory, antioxidant, regenerative, antimicrobial, and antibiofilm effects; and (iii) discuss their translational relevance, including advantages, limitations, oral-environment stability, mucosal retention, safety considerations, regulatory challenges, and future clinical perspectives. By integrating mechanistic insights with therapeutic and translational considerations, this review seeks to clarify the current landscape of curcumin nanoformulations and their potential role in advancing preventive and therapeutic strategies in dentistry. Given the complexity of the oral environment, which involves pH variations, the presence of saliva, enzymes, microbial biofilm, and rapid clearance, the selection of an appropriate nanoformulation is essential for achieving a sustained therapeutic effect.

To improve transparency regarding the scope of this review, it should be noted that the present manuscript was conceived as a narrative review rather than a formal systematic review. The literature considered for inclusion was prioritized on the basis of relevance to curcumin-based nanoformulations for oral health applications, with emphasis on studies addressing formulation design, physicochemical properties, molecular mechanisms of action, antimicrobial and antibiofilm effects, tissue-regenerative potential, and translational considerations. Priority was given to English-language in vitro, ex vivo, in vivo, and early clinical studies directly related to oral pathogens, oral biofilms, periodontal disease, oral mucosal conditions, and dental applications. Studies focused exclusively on native curcumin without a nanoformulation component, non-oral disease models without clear translational relevance to oral health, duplicate reports, or publications lacking sufficient methodological detail were not prioritized in the present review.

Beyond its relevance to oral biology and dental medicine, this review is also intended to provide practical value for scientists in pharmaceutical technology by critically integrating formulation design, carrier selection, physicochemical and biopharmaceutical constraints, release behavior, mucosal retention, manufacturability, safety evaluation, and translational considerations relevant to the development of clinically applicable curcumin delivery systems.

## 2. Types of Nanoformulations for Curcumin Delivery

**Figure 3:**
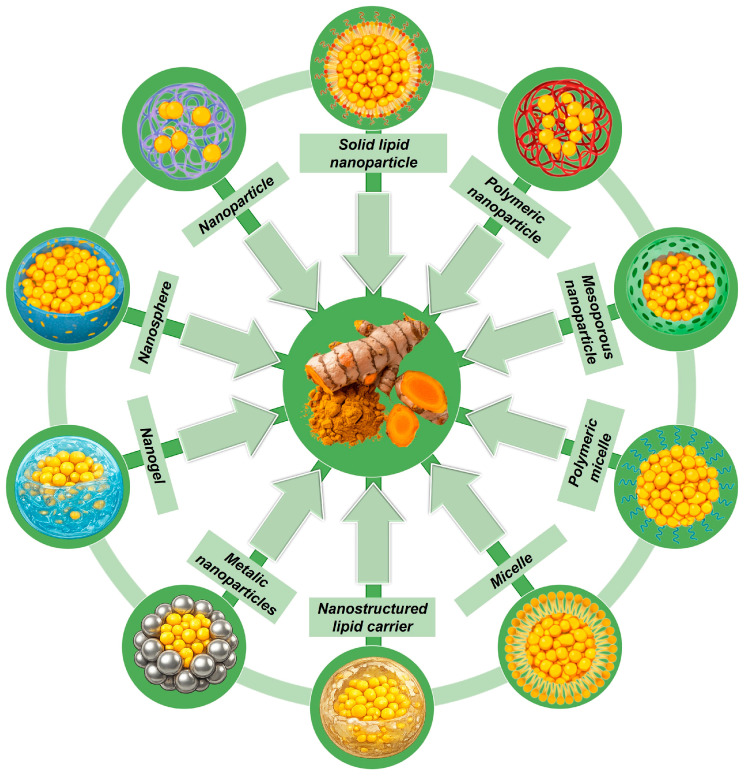
Schematic overview of the main curcumin-based nanoformulations investigated for oral health applications. Representative nanocarrier platforms include polymeric nanoparticles, solid lipid nanoparticles, nanostructured lipid carriers, micelles, nanogels, nanospheres, mesoporous nanoparticles, and metallic nanoparticles. These delivery systems are designed to improve curcumin solubility, stability, bioavailability, controlled release, and local retention, thereby enhancing its anti-inflammatory, antioxidant, antimicrobial, and antibiofilm potential in the oral environment.

The main classes of curcumin-based nanoformulations relevant to oral health applications are schematically illustrated in Figure 3.

### 2.1. Polymeric Nanoparticles

Polymeric nanoparticles are among the most extensively studied systems for curcumin delivery and are fabricated from biodegradable and biocompatible polymers such as poly(lactic-co-glycolic acid) (PLGA), poly-ε-caprolactone (PCL), polylactic acid (PLA), or chitosan. Owing to their versatility, these systems allow adjustment of particle size, loading degree, and active substance release rate. Biodegradable polymeric platforms (PLGA, PCL, PLA, and chitosan) have been widely investigated for curcumin delivery because they combine biocompatibility with prolonged release and protection of curcumin against degradation [56,57,58,59,60]. Polymers such as PLGA offer the advantage of controllable degradation and excellent compatibility with oral tissues and have already been approved for medical applications. Chitosan, a naturally occurring positively charged polymer, exhibits intrinsic mucoadhesive and antimicrobial properties, making it particularly attractive for antibiofilm applications. In combination with curcumin, polymeric nanoparticles may facilitate penetration into the superficial layers of the biofilm and may prevent its formation. For example, Ma and colleagues emphasize PLGA and chitosan in the context of nanoparticles for periodontal and biofilm-related applications [56]. Similarly, Ndumiso and colleagues present PLGA and PCL as biodegradable polyester nanoparticles and examine how their surfaces acquire a protein corona in serum, a factor that may influence stability, targeting, and interaction with biofilms [57]. Grilc and colleagues summarize various strategies for curcumin delivery, ranging from solid dispersions to nanoparticles and polymeric micelles (including PLGA/PEG-PLGA systems), and show how formulation parameters influence plasma exposure and, indirectly, tissue distribution [61]. In addition, the translational potential of curcumin nanoformulations is supported by preclinical findings and clinical studies, such as the randomized trial in patients with non-alcoholic fatty liver disease (NAFLD), together with pharmacokinetic data showing that various nanoformulations increase bioavailability compared with native curcumin [62].

The relevance of these systems falls within the broader context of nanomedicine, where biodegradable and biocompatible polymers (such as PLA/PLGA and related copolymers) act as carriers capable of high drug loading and controlled release. Agrahari and colleagues illustrate this through composite platforms based on biodegradable copolymers designed for the long-term release of macromolecules, in which the choice of polymer architecture determines formulation stability and release kinetics [58]. The block copolymers and surface engineering can modulate biodistribution, enable the crossing of biological barriers, and control release in response to environmental stimuli, principles that are directly applicable to the design of curcumin-loaded polymeric nanocarriers for the treatment of biofilm-associated infections [59,60].

#### 2.1.1. Advantages and Practical Implications of Using Polymeric Nanoparticles

- Solubility and systemic exposure

Curcumin-based polymeric nanoparticles allow substantial improvement in water solubility and, consequently, systemic bioavailability. The incorporation of curcumin into PLGA-based polymeric nanoparticles may lead to significant improvements in solubility, with major increases in water solubility demonstrated in vitro (including a reported 640-fold increase) and broader implications for bioavailability [56,63]. From a clinical perspective, nanocurcumin has been shown to improve metabolic, inflammatory, and hepatic function indices in patients with NAFLD, consistent with the improved bioavailability and increased tissue exposure of curcumin-based nanoformulations (including PLGA-based systems) in human subjects [62].

- Stability and loading capacity

Polymeric nanoparticles based on PLGA, chitosan, and PCL are highlighted for their stability in biological environments and for their ability to protect curcumin from degradation pathways (e.g., hydrolysis and oxidative processes) during storage (lyophilization and resuspension) and during in vivo testing [56,58,59,61,64]. The polymer composition (PLGA, PCL, and related polyesters) provides the chemical and physical stability required for storage and distribution while also allowing degradation and controlled release in physiological environments [58,59,60,64].

- Controlled and sustained release

A defining advantage of biodegradable polymeric nanoparticles is the possibility of tailoring release kinetics to achieve sustained exposure of curcumin at the target site [61]. The release behavior of curcumin from PLGA- and PCL-based systems can be adjusted through polymer composition, molecular weight, crystallinity, and particle morphology, allowing sustained therapeutic exposure that is advantageous for the treatment of chronic infections and biofilm-associated pathological conditions [56,58,60,61].

- Nanoparticle surface physics and implications of the protein corona

The interaction of polymeric nanoparticles with biological fluids is a key factor determining stability, biodistribution, and interaction with biofilms. Ndumiso and colleagues demonstrated that PLGA- and PCL-based polymeric nanoparticles acquire a protein corona in human serum, which may alter particle surface characteristics, cellular uptake, and biodistribution, factors that indirectly affect stability and release behavior in vivo and in biofilm environments [57].

#### 2.1.2. Mechanistic Considerations of Polymeric Nanoparticles Regarding Penetration into Oral Biofilm

Polymeric nanoparticles, including PLGA- and PCL-based nanoparticles, exhibit tunable dimensions and surface chemical properties that influence diffusion and interaction with biofilm matrices. It has been indicated that protein adsorption on the surface of polymeric nanoparticles modulates interactions with biological interfaces, including biofilms, and may therefore affect penetration and residence time within biofilms [57]. This is consistent with reports correlating surface engineering and protein corona dynamics with biofilm interactions [59,60]. Although not present in all curcumin-based nanoformulations, chitosan-containing nanosystems are often used because of their mucoadhesive potential and electrostatic interaction with negatively charged biofilms. Ma and colleagues highlight the inclusion of chitosan as a representative polymer in the periodontal context, suggesting that chitosan-containing nanoparticles may improve contact with biofilms and mucosal surfaces, a factor relevant to penetration into and retention within oral biofilms [56].

It has been shown that polymeric matrices may modulate bacterial adhesion and biofilm virulence. PLGA-based coatings for curcumin-loaded vascular stents demonstrate nearly linear curcumin release and improved hemocompatibility, illustrating the potential of these biodegradable films to stabilize and control local curcumin delivery on intravascular surfaces [65]. In the periodontal context, rapidly releasing polymeric nanofibers have been shown to significantly reduce mixed biofilm formation, underscoring the role of polymer architecture and composition in the control of oral biofilm [66]. Furthermore, Mahmoud and colleagues reported that polymeric platforms, including PLGA-based nanofibers and nanoparticles, can inhibit the adhesion of *Porphyromonas gingivalis* to *Streptococcus gordonii* and can modulate biofilm formation, highlighting the role of polymeric matrices in controlling microbial interactions at the biofilm level [66,67]. Similarly, Ma and colleagues review targeting of the Nrf2 pathway in periodontal disease and discuss the use of curcumin and its nanoformulations (including PLGA systems) as modulators of oxidative stress and inflammation, which supports the translational relevance of curcumin-loaded polymeric nanocarriers for biofilm-associated periodontal infections [56].

The plausible mechanism proposed on the basis of data from the literature is a multifactorial one for biofilm penetration and for the enhanced activity of curcumin-based polymeric nanoparticles in biofilms. This mechanism refers to the following:

- Diffusion-dominated transport through extracellular polymeric substances (EPS) is facilitated by nanoscale size and diffusion-favorable surfaces; smaller nanoparticles with a hydrophilic/hydrophobic balance may traverse EPS channels more easily than larger aggregates [59,60];
- Surface charge and interactions with the biofilm matrix modify residence time within the biofilm; positively charged or mucoadhesive surfaces (e.g., chitosan-containing systems) may favor initial contact and retention in the biofilm, increasing local exposure to curcumin [56];
- Protein corona effects modulate biofilm interactions and uptake pathways; corona-modified surfaces may alter binding to extracellular components, influence diffusion through biofilm layers, and affect microbial uptake of the nanoformulation [57];
- Drug loading and controlled release sustain antimicrobial exposure within the biofilm microenvironment, overcoming rapid clearance and allowing prolonged curcumin activity against biofilm-associated pathogens [56,58,61,63].

**Figure 4:**
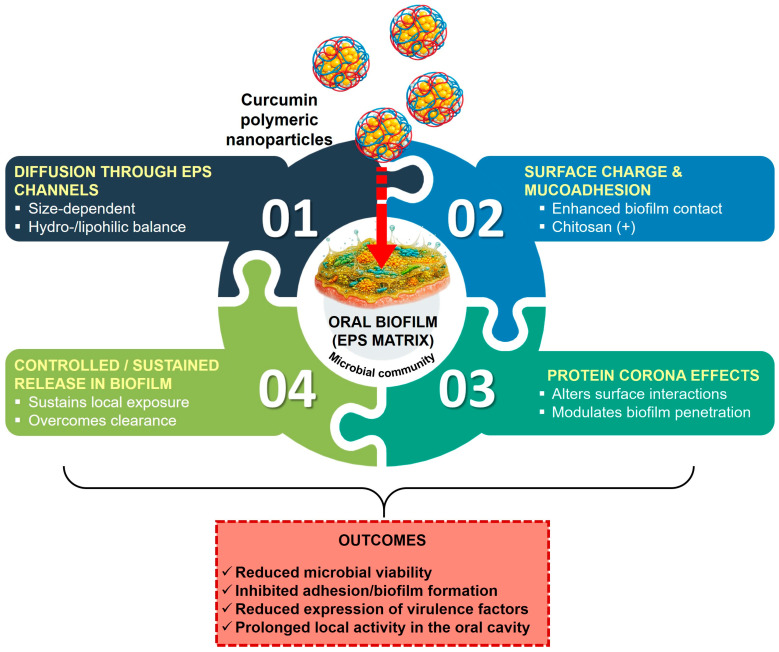
Proposed mechanisms of oral biofilm penetration by curcumin-loaded polymeric nanoparticles. Curcumin-loaded polymeric nanoparticles may interact with oral biofilms through diffusion across extracellular polymeric substance (EPS) channels, surface charge-dependent adhesion and mucoadhesion, protein corona-mediated modulation of surface interactions, and controlled or sustained drug release within the biofilm microenvironment. Collectively, these mechanisms may enhance local curcumin exposure, reduce microbial viability, inhibit adhesion and biofilm formation, suppress virulence factors, and prolong antimicrobial activity in the oral cavity.

The main mechanisms through which curcumin-loaded polymeric nanoparticles interact with and penetrate oral biofilms are schematically summarized in Figure 4.

Overall, the penetration of polymeric nanoparticles into biofilms, particularly in the oral environment, requires careful analysis of local delivery pathways, residence time, and surface properties. Evidence from the periodontal context (e.g., PLGA/curcumin systems affecting *P. gingivalis* interactions) indicates the potential of polymeric curcumin formulations to influence biofilms at their source, although detailed mechanistic characterization of diffusion through mature biofilms remains a distinct area of investigation [56,57,66].

#### 2.1.3. Challenges and Perspectives for Future Research

Some polymeric curcumin-based systems generated through polymerization may contain residual monomers, oligomers, or catalysts, which raise safety concerns and require rigorous purification and quality control for clinical application [63]. This aspect is important for PLGA- and chitosan-based nanoformulations intended for use in human subjects [56,63]. Furthermore, achieving high curcumin loading capacity while maintaining biocompatibility and favorable release kinetics remains a practical challenge, but it is a central theme in discussions on translational polymeric nanocarriers, which emphasize high drug-loading capacities and robust release profiles in PLGA, PCL, and related systems [58,68].

The interaction of polymeric nanoparticles with biological fluids alters surface characteristics and may influence biofilm interactions, distribution, and efficacy. Systematic exploration of corona effects on PLGA and PCL nanoparticles in the context of oral biofilm will help optimize design parameters for penetration and sustained activity within biofilms [57,69].

Overall, PLGA-, chitosan-, and PCL-based polymeric nanoparticles provide a coherent platform for curcumin delivery, enabling improved solubility, stability, and controlled release, with promising implications for biofilm-associated infections, in which targeted delivery and prolonged exposure are advantageous. Limitations may arise from the complexity of preparation methods (precipitation, emulsion-diffusion, controlled polymerization) or from possible variations in curcumin loading depending on the solubility of the compound in the phase used. Nevertheless, their efficacy makes them one of the most promising options in dentistry. Future research should quantify biofilm penetration mechanisms, optimize surface engineering for biofilm access, and address manufacturing and safety considerations in order to enable broader clinical translation.

### 2.2. Nanomicelles and Nanoemulsions

Nanomicelles and nanoemulsions represent another type of platform developed to overcome the limitations related to poor water solubility, rapid metabolism, and low oral bioavailability, which in turn restrict the clinical use of curcumin despite evidence of safety at high oral doses in humans [34,70]. Considerable efforts have therefore been devoted to the development of nanoformulations capable of solubilizing curcumin and enhancing systemic exposure, and nanomicelles and nanoemulsions have proven to be particularly versatile platforms when constructed from surfactants or amphiphilic polymers [51,71,72]. These nanoformulations are designed to encapsulate curcumin within a hydrophobic core, thereby protecting it from premature metabolism and enabling higher effective concentrations at the target site, either systemically or locally at mucosal surfaces [34,71].

Nanomicelles and nanoemulsions constitute two major nanoscale vehicles used for curcumin solubilization through distinct self-assembly processes. Nanomicelles (polymeric micelles or surfactant-based micelles) are formed when amphiphilic molecules arrange into a core–corona architecture in aqueous media, creating a hydrophobic core capable of loading curcumin and a hydrophilic shell that stabilizes the assembly in water [51,72]. In contrast, nanoemulsions are kinetically stable oil-in-water (O/W) dispersions stabilized by surfactants (and sometimes cosurfactants) that disperse hydrophobic curcumin within an oily phase distributed throughout a continuous aqueous phase, thereby increasing the apparent solubility and diffusion of curcumin [71].

#### 2.2.1. Advantages and Practical Implications of Using Nanomicelles and Nanoemulsions

The main advantages of these nanoplatforms are related to the solubilization and absorption provided by these micellar systems and nanoemulsions. It has been reported that a curcumin microemulsified system dramatically increases the intestinal absorption of curcumin and associated curcuminoids in animal models, with the reported enhancement factors being notable depending on the formulation components (e.g., capryol-based oily phases and nonionic surfactants) [71]. Nanoemulsions, stabilized with surfactants and with particle diameters below 200 nm, offer not only rapid absorption but also the advantage of visual clarity (when a transparent product is desired) and long-term stability [73,74]. Nanomicelles exhibit increased solubility in aqueous media and can be incorporated into mouthwashes, gels, or oral sprays [75,76]. Curcumin-based nanoemulsions have been developed and investigated, demonstrating a significant reduction in bacterial biofilm and oxidative stress [77,78,79]. Similarly, Al-Maweri and colleagues highlighted the efficacy of a curcumin nanoemulsion-based mouthwash, which reduced plaque index and gingival inflammation in patients, showing performance comparable to chlorhexidine but without its adverse effects [80].

Both systems exhibit enhanced biofilm penetration and the possibility of uniform release over the mucosal surface [81,82]. However, their stability depends on the type of surfactant used, and excessively high concentrations may raise concerns regarding cytotoxicity or may affect the taste of oral products [83,84,85].

Other studies report substantial improvements in the oral bioavailability of curcumin when administered as micellar formulations or nanoemulsions compared with conventional curcumin preparations, with improved pharmacokinetic profiles and tissue exposure [51,70]. Even in comparative human pharmacokinetic investigations, these nanoformulations (micelles, nanoemulsions, and related carriers) are identified as important strategies for overcoming the challenges associated with curcumin solubility and bioavailability [86,87,88].

Concerning practical implications, the topical/local activity potential of curcumin-based nanoformulations is particularly attractive for oral care applications, including mouthwashes and oral sprays, where direct mucosal exposure may benefit from solubilized and stabilized curcumin with anti-inflammatory and antimicrobial potential. Nanoformulations have been extensively studied for oral health, including gels and mucoadhesive delivery systems targeting oral submucous fibrosis and associated inflammatory conditions [89]. The same principles of nanoemulsions and micelles that enhance oral bioavailability at the systemic level may, in principle, be adapted to aqueous oral care formats, enabling stable curcumin dispersion in mouthwashes and sprays with controlled release or rapid dissolution while maintaining mucosal compatibility and therapeutic activity [89]. The literature emphasizes the practical aspects of translating nanoformulations into consumer-oriented products, including considerations related to stability, scalability, and regulatory pathways for curcumin delivery systems and their potential for clinical translation [51,88]. Overall, these findings support the strong potential for incorporating curcumin-based nanoformulations into mouthwashes and oral sprays as part of an oral health strategy, with mechanistic benefits resulting from improved solubility, mucosal exposure, and sustained anti-inflammatory action in the oral cavity [89].

#### 2.2.2. Mechanistic Considerations of Nanomicelles and Nanoemulsions

The rationale for designing nanomicelles and nanoemulsions is based on their ability to solubilize and protect hydrophobic curcumin within a hydrophobic core, thereby increasing its apparent solubility in aqueous media and enabling more favorable pharmacokinetics. In polymeric micelles, curcumin partitions into the hydrophobic core formed by the micellar structure, while the hydrophilic corona stabilizes the particle in water and may modulate interactions with biological interfaces, thereby increasing the dissolution rate and systemic exposure compared with free curcumin [51]. In the case of nanoemulsions, curcumin is located within oil droplets dispersed in the aqueous phase; surfactants stabilize the oil droplets, preventing coalescence and enabling transport through the intestinal epithelium, which results in greater oral bioavailability [71]. Therefore, solubilization within a hydrophobic domain is a key determinant of the improved pharmacokinetic performance of curcumin, as reflected in animal pharmacokinetic studies and in human pharmacokinetic studies comparing nanoformulations with conventional preparations [71,86].

The intrinsic mechanisms underlying curcumin action (multitarget anti-inflammatory signaling, antioxidant activity, modulation of transcription factors, cytokines, and prosurvival pathways) form the basis of its therapeutic potential in various diseases, including inflammatory conditions and cancer, and are preserved and often amplified when curcumin is administered through nanoformulations that improve bioavailability and tissue exposure [34,51,70]. Nanoformulations that increase systemic bioavailability do not alter the fundamental molecular targets of curcumin; rather, they enhance the magnitude and consistency of curcumin interaction with these targets by delivering a greater amount of the active molecule to relevant sites, potentially amplifying its anti-inflammatory and antioxidant benefits in vivo [34,51].

#### 2.2.3. Challenges and Perspectives for Future Research

Despite their promising performance, nanomicelles and nanoemulsions still face several limitations that may affect their translational applicability in oral health. One important challenge relates to their physical stability, particularly in diluted biological environments, where micellar systems may undergo disassembly near or below the critical micelle concentration, potentially leading to premature drug leakage and reduced therapeutic efficiency. In addition, the long-term stability of nanoemulsions may be influenced by droplet coalescence, Ostwald ripening, surfactant composition, and storage conditions, all of which may affect formulation reproducibility and shelf life.

Another important consideration concerns biocompatibility and formulation acceptability. Because these systems rely on surfactants and, in some cases, cosurfactants, excessive concentrations may increase cytotoxicity risk or negatively affect the taste, tolerability, and mucosal compatibility of oral products. Furthermore, although nanomicelles and nanoemulsions have shown improved solubility, bioavailability, and antibiofilm performance, direct comparative data on their long-term antimicrobial efficacy under clinically relevant oral conditions remain limited.

Future research should therefore focus on optimizing surfactant composition, strengthening formulation stability under salivary dilution and pH fluctuations, and improving reproducibility during scale-up. Additional studies in multispecies oral biofilm models, salivary simulation systems, and controlled clinical settings are needed to determine whether the favorable physicochemical and pharmacokinetic properties of these formulations can be translated into durable therapeutic benefits in the oral cavity.

### 2.3. Solid Lipid Nanoparticles (SLN) and Nanostructured Lipid Carriers (NLC)

Nanostructured lipid carriers, particularly SLNs and NLCs, have also proven to be promising candidates for overcoming the biopharmaceutical limitations of curcumin by improving its solubility, protecting it against degradation, enabling controlled release, and enhancing retention in biological environments [51,89,90,91]. SLNs and NLCs are recognized as complementary systems, with SLNs representing the first generation of delivery systems and NLCs the second generation, developed to combine the advantages of polymeric nanoparticles with those of lipid emulsions [89,90]. The combination of solid and liquid lipids in NLCs leads to reduced lipid crystallization, which favors higher drug loading and sustained release while maintaining biocompatibility, whereas SLNs are composed entirely of solid lipid matrices at body temperature, offering increased stability and prolonged drug release [89,90]. These nanoformulations have been extensively explored for oral and dental applications, providing a framework for discussing stability, release kinetics, mechanism of action, and translational potential in oral gels and toothpastes [51,89,90]. Formulations with optimized lipid ratios exhibit favorable release profiles and improve curcumin solubility, which is essential for maintaining therapeutic concentrations in the oral cavity [92]. NLCs often outperform SLNs in terms of drug loading and minimization of drug expulsion, thereby facilitating prolonged curcumin release in the oral environment [89,90].

Drug release from NLCs typically involves an initial burst release from the particle surface, followed by sustained release as the lipid matrix undergoes degradation or reorganization over time [93]. Sustained release in NLCs arises from their imperfect crystalline structure and the presence of liquid lipids, which create regions capable of accommodating curcumin, thereby effectively delaying its diffusion [89,90,94].

#### 2.3.1. Advantages and Practical Implications of Using SLNs and NLCs

The advantages and limitations of SLNs and NLCs for oral applications refer to the manner in which these formulations may improve curcumin bioavailability and enable targeted delivery to oral tissues [51,95,96,97].

The dynamic environment of the oral cavity, including salivary flow and enzymatic activity, presents a challenge for drug stability and retention. Although direct measurements of curcumin-NLC/SLN stability in saliva are limited, the literature indicates that curcumin-based nanoformulations provide improved stability against degradation compared with free curcumin [51,94,95]. This protective effect derives from the lipid matrix surrounding curcumin, contributing to sustained release in the salivary environment [94].

NLCs are particularly suitable for prolonging curcumin release due to their semicrystalline matrix, which allows controlled diffusion-based release and minimizes drug expulsion [90,94]. Experimental studies in NLC systems describe an initial burst release followed by slower matrix-based release correlated with lipid degradation, which supports the long-term availability of curcumin in the oral cavity, as well as in gel and paste formulations [93,94].

The stability and controlled-release characteristics of curcumin-loaded NLCs suggest advantageous integration into oral gels and toothpaste formulations. Hydrogel matrices may act synergistically with NLCs to enhance contact time with mucosal surfaces, and studies have demonstrated the incorporation of curcumin into hydrogel systems for dental applications [89,98,99]. Moreover, the integration of curcumin-loaded NLCs into hydrogel matrices has also been explored in dermatological and non-oral dental contexts, presenting co-formulations that combine the protection afforded by lipid nanoparticles with the adhesion and prolonged release provided by hydrogels [99,100]. Although few studies directly address curcumin-loaded NLCs in toothpaste compositions, the demonstrated efficacy of curcumin-loaded NLCs and SLNs in gel matrices suggests a promising path for translation into dentifrice formulations; these systems may enhance antimicrobial and anti-inflammatory effects through prolonged surface contact and gradual curcumin release [51,89,90,98]. In addition, curcumin-loaded NLCs have been investigated for local delivery to dental tissues and post-extraction healing, demonstrating their potential function as targeted drug delivery systems in oral applications [95,99,100].

Although the available data generally support improved curcumin stability and release in SLN/NLC formulations, significant gaps remain in direct salivary stability studies comparing curcumin-loaded NLCs and curcumin-loaded SLNs. Therefore, more detailed investigations are needed to quantify these protective effects and release kinetics in vivo [89,90,95].

#### 2.3.2. Mechanism of Curcumin Action Relevant to Oral Tissues and How Nanoformulations Modulate Activity

Curcumin exhibits anti-inflammatory, antioxidant, antiproliferative, and proapoptotic effects through modulation of multiple signaling pathways, including NF-κB, COX-2, cytokines such as TNF-α, MAPK, and regulators of apoptosis. These mechanisms underline the therapeutic potential of curcumin in inflammatory oral diseases, such as periodontitis [51,95,96,97]. Encapsulation of curcumin into SLNs and NLCs improves solubility, facilitates cellular uptake, and enhances biological activity in both in vitro and in vivo studies by increasing local curcumin concentrations in target tissues and protecting it against premature degradation [51,95,96,97]. Therefore, the improved stability of curcumin and its sustained release from lipid nanoparticles may prolong the modulation of inflammatory mediators and reactive oxygen species (ROS) in the oral cavity, thereby justifying the integration of curcumin-NLC/SLN systems into products focused on dental caries prevention, periodontal therapy, and mucosal healing [51,89,95,97,98].

#### 2.3.3. Challenges and Perspectives for Future Research

Further research is needed on the in vivo salivary stability and pharmacokinetic profiles of curcumin-NLC/SLN formulations to determine precisely their protective properties and release behavior in real saliva. Although the existing literature supports improved stability and release, specific investigations addressing salivary parameters would strengthen confidence in toothpaste- and gel-type formulations [89,90]. Future studies should focus on optimizing the interactions among curcumin, lipids, and polymers in oral gels to maximize mucosal adhesion and ensure uniform nanoparticle dispersion and reproducible release kinetics under salivary conditions [98,99,100].

Despite the biocompatibility advantages of curcumin-NLC/SLN systems, comprehensive toxicity assessments and long-term safety evaluations are essential for regulatory approval and consumer acceptance of oral products intended for daily use [48,90,99]. Additional research on co-encapsulation strategies, in which curcumin is combined with antimicrobial agents or fluoride in NLC/SLN matrices, could produce synergistic effects for oral health [48,90,94].

### 2.4. Nanogels, Hydrogels, and Mucoadhesive Films

Nanogels and hydrogels are three-dimensional structures capable of absorbing large amounts of water while maintaining structural stability. Mucoadhesive oral films represent a modern alternative, as they can be applied directly to the gingiva, buccal mucosa, or areas affected by lesions. These oral films prolong the contact time of curcumin with tissues, reduce its dispersion in saliva, and may incorporate multiple ingredients (probiotics, metallic nanoparticles, local anesthetics).

Curcumin-based hydrogels, nanogels, nanomicelles, and mucoadhesive films have been developed for local application in the oral cavity because curcumin possesses anti-inflammatory, antioxidant, and antiproliferative properties that support mucosal protection, wound healing, and chemoprevention in the oral environment. These nanoformulations allow increased local residence time and controlled drug release [101,102,103]. Like other nanosystems, hydrogels and nanogels improve curcumin solubility and modulate release kinetics, while mucoadhesive patches/films ensure mucosal residence time and improved dosing precision in the buccal/oral context [101,102,103,104]. The combination of a mucoadhesive film with nanoencapsulation supports sustained curcumin release directly at the level of the oral mucosa, improving efficacy in conditions such as radiotherapy-induced mucositis and mucosal inflammation [102,103]. Thus, nanoformulations may deliver curcumin more efficiently to the target mucosa, supporting both local anti-inflammatory actions and broader tissue-healing processes [102].

#### 2.4.1. Mucoadhesive Films and Patches for the Oral Cavity

The use of buccal/oral mucoadhesive patches and films is highly relevant for curcumin delivery in the oral cavity because of their flexible, patient-tolerated form, localized delivery capability, and controlled-release potential. Mucoadhesive patches provide flexible and better-tolerated alternatives to tablets and can efficiently deliver drugs to targeted mucosal areas, with improved dosing precision, localized effect, controlled release, and robust mucoadhesion [105,106,107].

The mucoadhesive mechanism of buccal patches involves rapid wetting and swelling of the polymer, which promotes close contact with the mucosa to enable adhesion, an essential condition for effective residence time and drug transfer to the buccal mucosa [106,107]. Moreover, studies addressing mucoadhesive buccal patches emphasize both the importance of adhesive interaction with the mucosa and the need to balance mucoadhesion with painless removal [108]. In the fabrication of functional films and mucoadhesive patches built from various polymers designed for buccal delivery, emphasis is placed on formulation strategies to optimize mucoadhesive strength, flexibility, moisture retention, and controlled drug release. For example, multifunctional mucoadhesive buccal patches have been developed using adhesive polymers and concomitant-release components, illustrating the feasibility of patch-based platforms for mucosal drug delivery [107]. Electrospun mucoadhesive membranes and related patch architectures demonstrate the versatility of mucoadhesive systems for oral medicine, including patch-based administration and large-surface mucosal contact [109]. The mucoadhesive patch design concept is further supported by studies on drugs delivered in buccal patches, including patches designed for chemoprevention or local therapy, which emphasize patch flexibility, patient tolerance, and precise dosing within the buccal mucosa. Therefore, mucoadhesive patches can be designed to accommodate various payloads and to provide localized and sustained release in the oral cavity [105,106,107,108,109].

Curcumin-loaded mucoadhesive films have been described in the literature as part of multifunctional biopolymeric composites (e.g., bacterial cellulose/alginate/gelatin matrices), in which the films remain adherent in the moist oral environment and allow sustained curcumin release at the mucosal surface, potentially benefiting antimicrobial and anticancer applications in the oral cavity [110,111]. In these systems, the mucoadhesive film maintains moisture and adheres to the buccal mucosa, releasing curcumin over time in accordance with the desired specific action in the oral cavity [110].

#### 2.4.2. Mucoadhesive Patches Loaded with Multiple Agents

A notable advantage of mucoadhesive patches and hydrogel/nanogel-based carriers is their ability to incorporate multiple bioactive agents into a single platform, thereby enabling combination therapies at the mucosal surface. Mucoadhesive patches and films have been designed to simultaneously release multiple active substances and to integrate additional functional components (e.g., antimicrobial or anticancer agents) into the same matrix, offering opportunities for synergistic effects at the oral mucosal level [106,107,108,109,112,113]. Curcumin-loaded biopolymeric films and patches have been used in various contexts, including bacterial cellulose-based films and alginate/gelatin composites, exhibiting sustained curcumin release and additional antimicrobial or anticancer activities in the oral/mucosal environment [110,111]. These formulations illustrate the feasibility of loading curcumin alongside other active substances into mucoadhesive matrices to achieve combined effects in the oral cavity [110,111]. In addition to curcumin and its concomitant delivery with other natural extracts or conventional drugs, mucoadhesive patches and hydrogels have been used for the simultaneous delivery of liposomes, nanoparticles, and other bioactive payloads, highlighting the versatility of the platform for multi-agent strategies that could include probiotics or silver nanoparticle-based antimicrobials within the same patch or in successive layers [114]. For example, curcumin-loaded liposomes incorporated into hydrogels have been explored for localized delivery in surgical or wound-treatment contexts, demonstrating the feasibility of combining curcumin with other nanoformulations within a single architecture [106,107,108,109,110,111,114,115].

#### 2.4.3. Practical Considerations and Mechanism of Action of Oral Patches with Curcumin Nanoformulations

The practical design of buccal patches involves creating flexible, thin films or patches that can adapt to irregular oral surfaces, ensuring controlled release while providing robust mucoadhesion without excessive discomfort or displacement. Mucoadhesive buccal patches have proven to be highly flexible and generally better tolerated than some tablet formulations, allowing controlled release and durable contact with the mucosa [105,106,107].

The adhesive mechanism of buccal patches is based on wetting, diffusion, and swelling processes that generate intimate contact between the patch and the mucosa, thereby establishing the basis for sustained drug release and mucosal residence time [106,107]. Patch fabrication methods include electrospinning to create mucoadhesive membranes with a large surface area and tunable mechanical properties, providing a support for curcumin-loaded fibers or films with prolonged mucosal contact [109]. Films and patches may be designed with backing layers and mucoadhesive matrices to optimize handling, adhesion, and drug release under the dynamic conditions of the oral cavity (salivary flow, tongue movement, moisture). For example, mucoadhesive patches often include a backing layer for unidirectional drug release and improved handling, while the mucoadhesive layer ensures mucosal adhesion and sustained curcumin release [105,106,107,108,109]. This multimaterial approach supports the design of curcumin nanoformulations into practical oral patches suitable for clinical use [105,106,107,109].

The therapeutic effects of curcumin on the buccal mucosa derive largely from its anti-inflammatory, antioxidant, and antiproliferative activities, which collectively contribute to reducing mucosal inflammation, protecting against oxidative stress, and promoting wound healing and mucosal recovery. In vivo evidence regarding the use of curcumin nanoformulations indicates enhanced systemic exposure and improved protection against mucosal injury (e.g., radiotherapy-induced mucositis), consistent with the anti-inflammatory and tissue-protective actions of curcumin in the oral environment [102]. This mechanistic action is supported by the activity of curcumin as a modulatory compound targeting inflammatory signaling and oxidative stress pathways relevant to mucosal health and healing [103,111].

Nanocarrier encapsulation improves curcumin stability and local concentration at the mucosal level, enabling more effective interaction with mucosal cells and tissues, which in turn may lead to amplification of anti-inflammatory and wound-healing effects in the oral cavity [102,103]. Curcumin-loaded biopolymeric films also exhibit sustained release and antimicrobial or antitumoral activities in the oral context, indicating a complex mechanism that supports mucosal protection and tissue repair when administered through mucoadhesive matrices [110,111].

Overall, the mechanism of action of curcumin nanoformulations in oral patches/hydrogels/nanogels centers on delivering higher local concentrations of curcumin to the mucosal surface, prolonging residence time, and enabling sustained modulation of inflammatory and healing pathways [102,103,110,111].

#### 2.4.4. Conclusions and Future Research Directions

Hydrogels, nanogels, and mucoadhesive patches represent promising nanoformulations concerning improving solubility, residence time, and localized therapeutic effects on the buccal mucosa, with tangible benefits demonstrated in the context of mucositis and mucosal healing [101,102,103]. Mucoadhesive patches provide a robust platform for the controlled, site-specific delivery of curcumin and potentially active substances (e.g., probiotics, silver nanoparticles, or other antimicrobial/anticancer agents) using compatible polymers and multilayer architectures to optimize adhesion and release kinetics [105,106,107,108,109,111]. These patch and hydrogel platforms demonstrate the feasibility of multi-agent payloads, thus providing the essential basis for such combination therapies [106,107,108,109,110,111,115].

Future research directions should address: (i) the systematic in vivo evaluation of multi-agent patches containing curcumin and probiotics or silver nanoparticles to establish synergistic effects on buccal mucosal health and infection control; (ii) optimization of patch composition and geometry for patient comfort, salivary dynamics, and convenient administration; and (iii) standardization of release kinetics and testing of mucosal adhesion to enable robust clinical translation.

### 2.5. Metallic or Hybrid Systems

Metallic and hybrid curcumin nanoformulations represent a coherent and increasingly validated approach for enhancing antibacterial and antibiofilm activity. The underlying concept is to exploit the bioactivity and solubility of curcumin together with the distinct physicochemical effects of metallic nanoparticles (particularly silver and, to a lesser extent, gold) to achieve superior antibacterial and antibiofilm performance. Hybrid structures, such as curcumin-coated or curcumin-activated silver nanoparticles (AgNPs), curcumin-loaded materials decorated with silver or gold, and core/shell or micellar systems integrating curcumin and metallic constituents, have demonstrated synergistic antibacterial effects compared with each component administered separately [116,117,118,119,120]. Curcumin nanoformulations with metal-based carriers may improve solubility, protect curcumin from degradation, and enable more efficient delivery to bacterial targets [121,122]. Metallic or hybrid curcumin nanoformulations may exhibit enhanced antibacterial and antibiofilm activity through cooperative mechanisms, including improved curcumin delivery, metal-induced membrane disruption, generation of reactive oxygen species (ROS), and metal ion-mediated enzymatic interference [117,118,119,120,121].

#### 2.5.1. Representative Nanoformulations and Their Characteristics

A wide range of nanoplatform types exists for curcumin delivery, including polymeric micelles, nanofibers, core–shell nanoparticles, and niosomes, all capable of simultaneously delivering curcumin and metallic components with enhanced antimicrobial performance [118,119,120]. The diversity of these systems highlights the versatility of curcumin as a functional component in hybrid nanomedicine, adapting release and targeting to specific pathogens [118,119]. A recurring theme is the improvement in curcumin solubility, stability, and bioavailability when it is incorporated into nanoformulations with metals or metal-containing carriers, repeatedly cited as a major factor underlying the improved antibacterial outcomes of these hybrid systems [121,123]. These improvements underpin practical antibacterial efficacy and pave the way for clinical translation, while also emphasizing the need for thorough safety evaluations [121,124,125].

Curcumin, used as a reducing/stabilizing agent under alkaline conditions, mediates the formation of silver nanoparticles (AgNPs) through green synthesis and forms complexes such as Ag(curcumin)+, highlighting the chemical interaction between curcumin and silver during nanoparticle formation and establishing a basis for curcumin–silver hybrid systems [116]. Such synthesis strategies underlie many hybrid formulations in which curcumin participates in nanoparticle formation and coating, thereby integrating the biological activity of curcumin into the metallic structure [116,126].

Curcumin-loaded nanostructures containing AgNPs have demonstrated enhanced antibiofilm effects against clinically relevant bacteria, addressing the limitation that curcumin alone often shows limited antibiofilm efficacy when administered as a free molecule [117]. This synergy has been attributed to the simultaneous disruption of bacterial membranes by the silver components and the improved delivery/retention of curcumin within the biofilm environment provided by the nanoformulation [117].

Hybrid systems comprising silver-decorated polymeric micelles loaded with curcumin have demonstrated superior antibacterial performance, attributed to membrane damage caused by the silver component combined with synchronized curcumin release triggered by bacterial lipases, thereby facilitating localized curcumin availability at the site of action [118]. This feature highlights the cooperative effect of metallic decoration and curcumin release dynamics on antibacterial efficacy [118].

Electrospun core/shell membranes loaded with curcumin@Ag have exhibited synergistic antibacterial properties, with AgNPs promoting singlet oxygen production and curcumin contributing its own antimicrobial actions. It has been shown that the presence of AgNPs enhances photodynamic pathways, increasing the antibacterial outcome under appropriate excitation or light conditions [119]. This feature illustrates how a fibrous membrane platform can integrate curcumin and AgNPs to achieve superior activity [119].

In niosomal formulations, curcumin-loaded vesicles containing AgNPs or copper nanoparticles (CuNPs) have shown enhanced antibacterial effects, with the combination being more effective against biofilms than each component alone; electron microscopy analyses confirming cooperative disruption of biofilm integrity by the two-component system [120]. This feature illustrates the potential of nanocarrier platforms to deliver synergistic metal–curcumin formulations to microbial communities [120].

Curcumin-promoted AgNP synthesis has used curcumin to stimulate the formation of AgNPs that subsequently self-assembled with a thermoresponsive polymer into core–shell nanohybrids, creating a modular hybrid system in which curcumin participates in both reduction and templating [126]. This pathway highlights how curcumin may enable the integration of metallic motifs into smart polymer environments [126].

Beyond Ag, curcumin has been conjugated with gold nanoparticles to explore potential antioxidant and antimicrobial behavior, demonstrating the broader compatibility of curcumin with noble metal nanoparticle platforms [127]. Although silver occupies a prominent place in the literature on antibacterial hybrid systems, curcumin-functionalized gold nanoparticles demonstrate the feasibility of silver-free metallic platforms, suggesting additional pathways for tuning antibacterial and antioxidant properties through metal–curcumin conjugation. This broadens the design space for metallic or hybrid curcumin nanoformulations [127].

#### 2.5.2. Mechanisms Underlying Synergy and Superior Antibacterial Activity

The synergy between curcumin and metallic nanoparticles, especially silver, arises from complementary mechanisms: improved curcumin delivery and local availability, metal-induced membrane disruption, generation of ROS and singlet oxygen, and simultaneous interference with bacterial enzymes and genetic targets. Silver nanoparticles are well-known antibacterial agents that exert their effects through the release of Ag+ ions, interaction with thiol groups in bacterial proteins, disruption of DNA replication, and induction of membrane damage [122]. When combined with curcumin, additional synergistic pathways emerge, including improved curcumin delivery to bacteria, membrane disruption, and potentiation of oxidative mechanisms [118,119,120].

In silver-decorated micelles carrying curcumin, the enhancement of antibacterial properties is attributed to the membrane-disrupting action of silver together with bacteria-triggered synchronized curcumin release, which increases the local curcumin concentration at the bacterial interface [118]. A similar rationale applies to AgNP-decorated nanofibers, where Ag-mediated membrane damage and the antimicrobial activity of curcumin act in concert under light conditions [119]. Moreover, AgNPs may promote singlet oxygen generation in curcumin-containing systems, leading to increased oxidative stress on bacteria and improved antibacterial outcomes; core/shell nanofibrous membranes integrating Curcumin@Ag exhibit superior activity, partly due to metal-enhanced singlet oxygen production, particularly under light exposure [119]. The challenges associated with curcumin solubility and stability are mitigated in nanoarchitectures that combine curcumin with silver-, gold-, or silica-based carriers; such platforms improve bioavailability and support release, enabling more effective interactions with bacterial targets than free curcumin alone [121,123]. Improved solubility and targeted release underlie the observed superiority of many curcumin–metal hybrids compared with the individual components [121,123].

In several systems, curcumin acts as a reducing and/or stabilizing agent in AgNP formation, enabling intimate integration of curcumin with metallic cores and facilitating subsequent antimicrobial action through colocalized activity; this dual role explains the robust performance of curcumin–metal hybrids in antibiofilm and antibacterial assays [116,126]. Gold–curcumin conjugates illustrate how metal–curcumin conjugation may preserve antioxidant properties and potentially extend antimicrobial functionality [127].

#### 2.5.3. Potential Risks and Safety Considerations: Cytotoxicity and Systemic Accumulation

Cytotoxicity toward host cells and systemic exposure are key issues that must be considered for metal–curcumin nanoformulations. Although curcumin itself is generally regarded as having relatively favorable biocompatibility, the addition of metallic nanoparticles, particularly silver, raises potential risks of cytotoxicity and systemic accumulation that require careful evaluation. Some studies emphasize biocompatibility considerations and the protective role of curcumin when combined with metal oxides, suggesting that metal-associated toxicity may be mitigated through formulation design and the use of biocompatible carriers, but this remains critical for safety assessment in translational applications [121,122].

The antimicrobial activity of silver is largely related to Ag+ release and interactions with thiol-containing biomolecules; however, this bioreactivity may also induce cytotoxic effects in mammalian cells, underscoring the need for controlled-release strategies and targeted delivery in hybrid formulations [122]. In composite systems, the bioactivity of silver must be balanced against potential systemic exposure and accumulation, particularly for in vivo applications [128]. The synergistic antibacterial benefits of curcumin–silver systems are compelling, but safety data specific to these hybrids remain limited. Reviews of curcumin nanoformulations with metal oxide-based materials emphasize the protective role that curcumin may play in mitigating metal oxide-associated toxicity [121]. Additional studies on systemic distribution, long-term accumulation, and organ-specific toxicity are needed to fully characterize the safety of these hybrid platforms [121,122].

The risks of systemic accumulation are influenced by particle size, surface chemistry, and carrier material; several studies show that the in vivo fate of silver- and curcumin-containing nanostructures depends on the formulation and route of administration. Moreover, complex biodistribution and elimination profiles that challenge direct clinical translation have been documented [128]. Broader reviews of curcumin nanoformulations also highlight the need for cautious long-term safety and pharmacokinetic evaluation as these platforms advance toward clinical contexts [121,124,125].

Overall, the integration of curcumin with metal-based carriers remains a promising strategy for overcoming its intrinsic limitations while exploiting metal-mediated antimicrobial mechanisms, provided that toxicity and accumulation risks are managed through rigorous design and evaluation [121,124,125]. Potential cytotoxicity and systemic accumulation remain important considerations for translational development, requiring careful safety assessment, biocompatible design strategies, and a thorough understanding of pharmacokinetics to balance efficacy with patient safety [121,122,128]. Future research should emphasize standardized comparative analyses, safety profiling, and mechanism-focused studies to optimize these promising hybrid platforms for clinical antimicrobial applications [121,124,125].

### 2.6. Comparative Advantages and Limitations of Representative Types of Nanoformulations for Curcumin Delivery

Curcumin nanoformulations encompass a broad range of carriers aimed at addressing the well-known problems related to curcumin solubility, stability, and bioavailability, while also enabling targeting and controlled release. However, these platforms differ substantially in their physicochemical behavior, loading capacity, release characteristics, antimicrobial performance, scalability, and translational maturity. For this reason, a comparative analysis of their major advantages and limitations is essential for understanding their relative suitability for oral health applications. In the case of polymeric nanoparticles, nanomicelles and nanoemulsions, solid lipid nanoparticles and nanostructured lipid carriers (NLCs), nanogels/hydrogels/mucoadhesive films, and metallic or hybrid nanosystems, the literature consistently demonstrates improved solubility and/or protection against degradation, enhanced permeability and tissue/cellular uptake, as well as the potential for sustained or targeted release compared with free curcumin. Scale-up and industrial translation are most advanced for polymeric and lipid-based nanoparticles, with reported scale-up strategies. In contrast, many metallic or hybrid nanosystems remain at early stages of development, with relatively greater complexity in terms of synthesis and scale-up. The degree of technological maturity varies across formulations: polymeric systems, lipid-based systems (including SLNs/NLCs), and certain hydrogel/gel-based systems are relatively mature, whereas certain hybrid/inorganic nanosystems remain at the preclinical stage but offer compelling multimodal capabilities. These patterns are supported by research on curcumin nanoformulations and their physicochemical and biological performance [51,129,130], as well as by studies focused on specific carriers and scale-up considerations [53,131,132,133,134,135,136,137,138,139,140,141,142,143,144,145]. From a clinical perspective, the relative value of these systems depends not only on their ability to improve curcumin solubility and bioactivity but also on their capacity to remain stable in the oral environment, provide controlled or sustained release, and maintain adequate retention at mucosal or periodontal surfaces despite salivary clearance and pH fluctuations.

Beyond the formulation-specific advantages and limitations discussed above, several cross-cutting challenges remain relevant across all classes of curcumin nanoformulations. These include the need for reproducible large-scale manufacturing, improved stability in the dynamic oral environment, better control of release behavior and mucosal retention, harmonized physicochemical characterization, and more robust safety assessment in oral tissues. In addition, the comparative translational value of different carrier types remains difficult to establish because many published studies use heterogeneous experimental designs, different microbial models, and non-uniform outcome measures. Future work should therefore prioritize standardized evaluation frameworks, clinically relevant oral biofilm models, and direct comparative studies capable of linking formulation properties with therapeutic performance and translational feasibility.

To improve understanding of the clinical applications, Table 1 presents a comparative summary of the advantages and limitations of the main types of nanoformulations for curcumin delivery.

Overall, polymeric nanoparticles offer good permeability, high stability, and controlled release, but can have higher production costs. Nanomicelles and nanoemulsions demonstrate excellent antimicrobial effects and stability, enhance permeability and bioavailability with moderate scalability. Solid–lipid nanoparticles (SLN) and nanostructured lipid carriers (NLC) provide good stability and biocompatibility but may face manufacturing challenges and slightly lower technological maturity. Nanogels, hydrogels, and mucoadhesive films improve drug loading and bioadhesion and have excellent biocompatibility, but may exhibit variability in performance due to their mechanical properties and face limitations in industrial scalability. At the same time, metallic or hybrid nanosystems exhibit strong and unique antimicrobial effects and enhanced cell viability, being tailored for specific applications, but raise toxicity concerns and often face issues related to regulatory approval alongside stability issues, varying technological maturity, and higher production costs.

## 3. Molecular Mechanisms of Action in Oral Health

**Figure 5:**
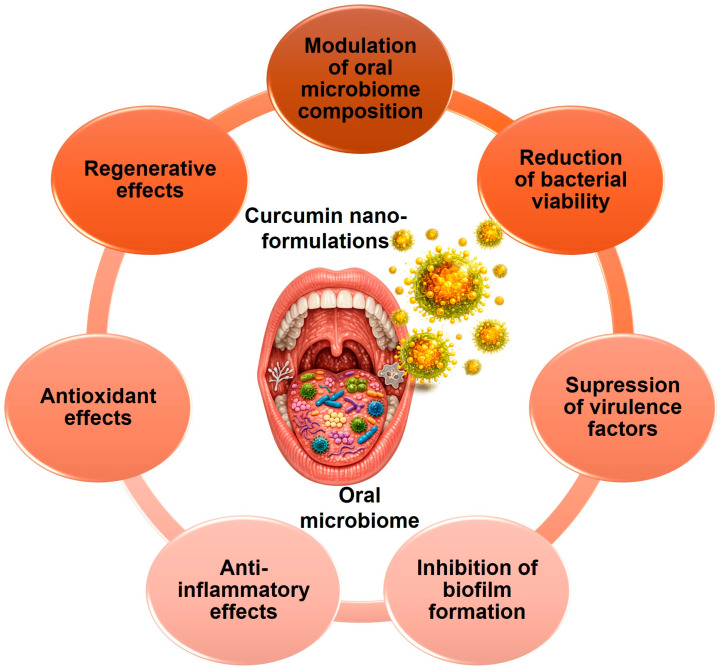
Schematic overview of the main biological effects of curcumin nanoformulations on the oral microbiome and oral tissues. Curcumin-based nanoformulations may suppress NF-κB-mediated inflammatory signaling, activate the Nrf2/HO-1 antioxidant axis, reduce oxidative stress, inhibit biofilm formation, decrease microbial viability and virulence, and support tissue repair and regenerative responses. Collectively, these mechanisms contribute to the therapeutic potential of curcumin nanoformulations in oral health applications.

Curcumin has attracted considerable interest in oral health due to its anti-inflammatory, antioxidant, and regenerative properties. When formulated as nanoscale delivery systems, curcumin bioavailability, tissue targeting, and efficacy in the gingival and periodontal context are improved, enabling more robust modulation of inflammatory pathways, redox homeostasis, and tissue regeneration. Curcumin-based nanoformulations suppress key inflammatory signaling cascades, activate antioxidant defenses through nuclear factor erythroid 2–related factor 2 pathways, and promote cellular functions relevant to periodontal regeneration. Accordingly, this chapter briefly describes the main mechanistic axes of curcumin-based nanoformulations, namely anti-inflammatory activity, antioxidant activity, and effects on tissue regeneration, with emphasis on their relevance to oral health, particularly gingivitis and periodontitis. Moreover, based on the anti-inflammatory, antioxidant, and regenerative actions of curcumin in the oral cavity, the most representative curcumin-based nanoformulations are increasingly being used to interact with oral biofilms and to reprogram local immune responses. Therefore, this chapter expands the molecular mechanisms of action of curcumin-based nanoformulations by integrating (i) how curcumin-loaded nanocarriers disrupt the bacterial extracellular matrix, influence initial bacterial adhesion, and enhance penetration into biofilms, as well as (ii) how these nanoformulations modulate resident immune cells to attenuate chronic periodontal inflammation. The principal biological effects of curcumin-based nanoformulations on the oral microbiome and oral tissues, including suppression of NF-κB-driven inflammatory signaling and activation of Nrf2/HO-1-associated antioxidant defenses, are summarized in Figure 5.

### 3.1. Anti-Inflammatory Activity

The central mechanism by which curcumin-based nanoformulations attenuate periodontal inflammation involves inhibition of the NF-κB signaling axis, a major regulator of proinflammatory gene expression in gingival tissues and in sites affected by periodontal disease. Curcumin consistently reduces NF-κB activation and its downstream transcriptional activity in models of bacterial lipopolysaccharide stimulation and inflammatory cytokine production, with reductions in interleukin-1 beta, interleukin-6, and tumor necrosis factor alpha reported across multiple systems. This pattern of action is reinforced by observations showing that curcumin reduces COX-2 expression and prostaglandin E2 production, both important mediators of periodontal inflammation and matrix degradation. The combined effect on these mediators, NF-κB, COX-2, and prostaglandin E2, is consistent with reduced gingival inflammation and diminished tissue destruction characteristic of gingivitis and periodontitis. Nanoformulations appear to potentiate these effects by improving curcumin bioavailability and local tissue concentrations at the gingival and periodontal levels, thereby reinforcing the downregulation of NF-κB signaling and its associated cytokine milieu. The anti-inflammatory profile of curcumin nanoformulations is supported by the observation that transcription factors (NF-κB and signal transducer and activator of transcription 1) are inhibited by curcumin, with concomitant decreases in interleukin-1 beta, interleukin-6, and tumor necrosis factor alpha across diverse inflammatory models [146,147,148,149,150,151]. In the human gingival context, curcumin nanoformulations reduce lipopolysaccharide solution (LPS)-induced proinflammatory cytokine production by gingival fibroblasts and suppress NF-κB activation, a mechanistic pattern compatible with clinical improvements in periodontal inflammation [146,152,153,154]. The anti-inflammatory actions of curcumin converge on kinases such as MAP kinases and on NF-κB signaling through inhibition of IκB kinase activity and IκB alpha degradation, thereby preventing NF-κB nuclear translocation and transcriptional activation of inflammatory genes [155,156,157,158,159].

In the anti-inflammatory context, the relevance to oral health is clear: by reducing NF-κB-driven transcription of proinflammatory cytokines (e.g., interleukin-1 beta, interleukin-6) and mediators such as COX-2 and prostaglandin E2, curcumin nanoformulations may attenuate the inflammatory cascade that drives gingivitis and periodontitis. Preclinical and clinical studies indicate curcumin’s ability to attenuate inflammatory mediators, including interleukin-1 beta, interleukin-6, tumor necrosis factor alpha, and COX-2, while increasing anti-inflammatory mediators such as interleukin-4 and interleukin-10; these effects are amplified when curcumin is delivered through nanoformulations, which improve bioavailability and therapeutic concentrations in periodontal tissues [146,147,160,161]. Discrepancies among studies regarding the exact transcriptional coregulators involved (e.g., interaction with peroxisome proliferator-activated receptor gamma and liver X receptor alpha) highlight a nuanced picture in which NF-κB remains a common node of curcumin action, while additional modulatory pathways may contribute to the anti-inflammatory outcome in periodontal contexts [147,150,152,162].

### 3.2. Antioxidant Activity

Curcumin-based nanoformulations also strengthen antioxidant defenses in oral tissues through activation of the nuclear factor erythroid 2-related factor 2 pathway, which coordinates cellular responses to oxidative stress. Activation of the nuclear factor erythroid 2-related factor 2 pathway leads to the upregulation of heme oxygenase-1 and other antioxidant enzymes, thereby contributing to the reduction of ROS in gingival fibroblasts and periodontal cells. This antioxidant axis is reinforced by curcumin’s ability to attenuate oxidative damage in inflamed environments and by the fact that curcumin may interact with the NF-κB signaling axis to limit oxidative stress-related inflammation. It has been shown that curcumin and certain nanoformulations activate the nuclear factor erythroid 2/heme oxygenase-1 axis, thereby reducing intracellular reactive oxygen species and protecting gingival and periodontal cells from oxidative injury. In addition, bacterial and inflammatory sources of reactive oxygen species are attenuated by anti-inflammatory actions that reduce neutrophil infiltration and the associated oxidative burst, complementing the direct antioxidant effects of natural product-derived compounds administered in nanoformulations [154,163,164,165,166].

Clinically relevant implications arise from the integration of these redox and inflammatory pathways. Periodontal tissues are subjected to oxidative stress during disease progression because of microbial challenges and host inflammatory responses; curcumin nanoformulations attenuate this stress both by activating nuclear factor erythroid 2-dependent antioxidant programs and by reducing NF-κB-driven pro-oxidant cascades. The net effect is a decrease in reactive oxygen species levels, reduced oxidative damage to gingival fibroblasts and periodontal cells, and restoration of redox balance in diseased sites [154,163,164,165,166].

### 3.3. Effects on Tissue Regeneration

In addition to their anti-inflammatory and antioxidant actions, curcumin-based nanoformulations promote tissue regenerative processes relevant to periodontal repair and regeneration. Curcumin may stimulate collagen production in gingival tissues and enhance gingival fibroblast proliferation, thereby contributing to the restoration of connective tissue integrity following inflammatory injury. Moreover, curcumin exerts pro-osteogenic effects on osteoblasts, supporting new bone formation and offering potential utility in periodontal regenerative strategies aimed at preserving or restoring alveolar bone. In nanoformulations, improved bioavailability and targeted delivery to periodontal tissues potentiate these regenerative signals by increasing local concentrations that stimulate collagen synthesis and fibroblast proliferation. The pro-regenerative effects on osteoblasts and periodontal ligament cell function are linked to broader evidence that curcumin and curcumin-based nanoformulations may influence osteogenic markers and extracellular matrix production, in alignment with the goals of periodontal regeneration [153,167,168,169,170,171].

### 3.4. Interaction with Oral Biofilms and Bacterial Cellular Structure

A central objective of curcumin-based nanoformulations in the oral environment is to disrupt the pathogenic biofilms that underlie gingivitis and periodontitis. The extracellular matrix of oral biofilms, which is rich in polysaccharides, proteins, and extracellular DNA, provides structural integrity and protection against antimicrobial agents. Curcumin delivered through nanocarriers such as solid lipid nanoparticles, liposomes, nanomicelles, and nanoemulsions has demonstrated the ability to compromise this matrix, thereby reducing biofilm robustness and facilitating antimicrobial penetration through improved solubility, stability, and tissue penetration, with downstream effects on biofilm matrix disruption and bacterial viability [172,173,174,175]. For example, curcumin has been shown to attenuate biofilm biomass and viability in *Streptococcus mutans* biofilms, with reductions in extracellular polysaccharide production and downregulation of glucosyltransferase B and glucan-binding proteins that drive adhesion and EPS network formation; in the nanoformulation context, this is associated with greater exposure of bacterial cells and more pronounced disruption of biofilm structure than free curcumin [78,174,176,177,178]. In addition, local administration of curcumin-loaded nanoparticles in periodontal disease models has led to marked attenuation of inflammatory bone resorption and inflammatory infiltration, consistent with reduced bacterial burden and diminished matrix-mediated protection of pathogens within the biofilm niche [179]. In this context, nanoparticle carriers may physically localize curcumin at the biofilm–tissue interface, increasing local concentrations at the site of bacterial colonization and enabling more effective disruption of biofilm structure [179]. Accordingly, nanocarriers enhance the antibiofilm activity of natural products by increasing local concentration, promoting sustained release, and improving penetration into the matrix and cells within the biofilm [175,180]. Thus, nanocarrier-facilitated delivery of curcumin improves penetration into heterogeneous biofilm architectures, enabling it to reach bacterial cells and biofilm-associated matrices that are less accessible to free curcumin formulations [175,179,181,182,183].

Initial bacterial adhesion to the tooth surface is a critical step in plaque formation. Nanoformulations may interfere with this step by delivering curcumin directly to microbiologically permissive surfaces and competing niches, thereby reducing the initial attachment of periodontal pathogens such as *Porphyromonas gingivalis* and *Aggregatibacter actinomycetemcomitans*. Although most studies focus on inflammation and bone resorption, the observed reductions in gingival inflammation and tissue destruction in animal models imply concomitant suppression of bacterial colonization, which would be expected to occur with decreased initial adhesion and improved disruption of early biofilm formation. This statement is supported by the anti-inflammatory and anticatabolic environment created by curcumin-based nanoformulations, which would reduce the pro-adhesive and pro-biofilm ambient signals generated by exposure to bacterial lipopolysaccharides and inflammatory mediators [179,184,185].

Penetration of curcumin into biofilms when delivered through nanocarriers has direct mechanistic implications. Nanocarriers may facilitate sustained release and intimate contact with the biofilm matrix, allowing prolonged exposure of biofilm-embedded bacteria to curcumin and its antimicrobial effects. In periodontal disease models, the ability of locally administered curcumin-loaded nanoparticles to inhibit inflammation and bone resorption correlates with the notion of improved local drug delivery within the periodontal microenvironment, including biofilm regions that are otherwise resistant to conventional antimicrobials [179,181]. Although direct microbiological quantification within biofilm matrices remains limited, the convergence of biofilm disruption and reduced inflammatory signaling strongly suggests that nanocarrier-mediated curcumin penetration contributes to attenuation of biofilm-associated pathogenicity in the oral cavity [179,181,184].

In addition to these general antibiofilm effects, photodynamic approaches using curcumin as a photosensitizer further exploit the advantages of nanoformulation. Curcumin encapsulated in nanocarriers or formulated as nanoemulsions may be activated by blue light to generate reactive oxygen species, causing direct damage to bacterial cells and matrix components. Recent in vitro and in situ studies have demonstrated improved photodynamic efficacy against *Streptococcus mutans* and *Candida* species when curcumin is delivered through nanoformulations or carrier systems that promote deeper penetration and sustained presence within the biofilm, leading to greater reductions in viability and disruption of biofilm architecture compared with non-encapsulated curcumin [175,177,182,183,186]. Moreover, studies on *Candida albicans* biofilms indicate that curcumin–sophorolipid nanoconjugates may inhibit filamentation and biofilm maturation, a key determinant of virulence that reinforces biofilm resistance; such nanoconjugates exhibit superior antibiofilm activity compared with free curcumin across multiple experimental models [175,187]. The efficacy of curcumin photodynamic therapy depends on efficient curcumin delivery and activation of the photosensitizer within the biofilm. Nanocarrier systems and nanogels have been shown to improve curcumin localization and retention within biofilms, thereby enhancing photodynamic inactivation when combined with adequate light exposure. However, variables such as biofilm maturity, photosensitizer localization, pre-irradiation time, and light dose critically influence outcomes; some studies report reduced efficacy in mature biofilms or under suboptimal light parameters, underscoring the need for optimized delivery–activation regimens in oral applications [175,182,186,188,189].

These multitarget actions are consistent with the broad-spectrum antibiofilm properties of curcumin, in which encapsulation or complexation improves delivery to the biofilm and modulates the expression of genes related to biofilm formation and virulence [173,174,178,180,190]. In the context of oral biofilms, these effects translate into reduced adhesion, diminished EPS production, and impaired maturation of multispecies biofilms, collectively attenuating cariogenic and periodontopathogenic biofilms in vitro and in some in vivo models [78,174,176,177,178].

### 3.5. Modulation of the Local Immune Response in the Oral Cavity

Curcumin-based nanoformulations not only suppress the factors that drive pathogen-associated biofilm formation but also modulate the local immune environment, which is essential in counteracting chronic periodontal inflammation. The local immune milieu in gingival tissues comprises resident macrophages and infiltrating immune cells that may adopt either proinflammatory M1 or anti-inflammatory M2 phenotypes; sustained M1 polarization contributes to chronic periodontitis through persistent production of tumor necrosis factor-α, interleukin-1β, and interleukin-6, thereby driving NF-κB-dependent inflammatory cascades. Curcumin-based nanoformulations exert anti-inflammatory actions consistent with reduced NF-κB activation and decreased proinflammatory cytokine levels in gingival tissues, in agreement with the observed reductions in proinflammatory cytokines in periodontal models treated with these nanoformulations [78,179,181,184,185].

A shift in macrophage polarization toward the M2 phenotype has been proposed as a mechanism through which curcumin-based therapies promote resolution of chronic inflammation and support tissue repair in the periodontal context. Although direct demonstrations of M2 polarization in gingival tissues following administration of curcumin-based nanoformulations are less abundant than findings focused on NF-κB, converging studies indicate that curcumin may promote M2-like characteristics and attenuate the production of M1-associated cytokines. In systems relevant to oral health, the net effect would be a reduction in chronic periodontal inflammation and restoration of tissue homeostasis, accompanied by improved preservation of the extracellular matrix and enhanced potential for periodontal regeneration [179,184,185,191,192,193].

In models in which macrophage-specific signaling modulates disease progression, downregulation of NF-κB signaling and the consistent decrease in tumor necrosis factor alpha and interleukin-1 beta are associated with reduced periodontal tissue destruction. Curcumin’s ability to attenuate proinflammatory signaling is consistent with reduced presence and activity of M1 macrophages in inflamed gingival tissues, thereby contributing to an immune environment more conducive to healing. A pro-resolving, anti-inflammatory environment, characterized by increased interleukin-10 and other regulatory mediators, supports the transition toward tissue repair in periodontitis, a transition potentially facilitated by curcumin-based nanoformulations through macrophage repolarization [194,195,196]. The concept of macrophage polarization as a therapeutic target in periodontal disease is supported by studies describing the dynamic M1/M2 balance in periodontitis and the potential to direct this balance toward repair and homeostasis. Periodontal inflammation is sustained by a predominance of M1-type macrophages, and strategies that shift polarization toward M2-like phenotypes may attenuate inflammation and promote regeneration. Curcumin-based nanoformulations, by delivering curcumin directly to inflamed sites and by modulating redox-sensitive signaling networks, fit within this host-modulation paradigm and may contribute to a more favorable immunological environment in the periodontium [191,192,193,196,197,198,199,200,201].

### 3.6. Potential Discrepancies Regarding the Effects of Curcumin-Based Nanoformulations

Although substantial evidence supports NF-κB inhibition and Nrf2 activation as the principal mechanisms of curcumin-based nanoformulations, some mechanistic findings remain heterogeneous across studies. The extent to which these pathways translate into clinical improvements in gingivitis versus periodontitis may vary according to formulation type, dosage, route of administration (local vs. systemic), experimental model, and the degree to which mechanistic endpoints are directly demonstrated in oral tissues rather than inferred from non-oral systems. Local nanoformulations often produce stronger suppression of inflammatory bone resorption and tissue infiltration in periodontal disease models compared with systemic administration, reflecting higher local drug concentrations and targeted deposition in gingival/periodontal tissues [179,181,184,185]. Although Nrf2 activation is well established in multiple cell types, direct mechanistic demonstrations in gingival or periodontal cells (e.g., PKCδ-mediated p62 phosphorylation leading to Nrf2 stabilization) require additional investigation specifically within oral tissues. Extrapolation from neuronal or other tissue systems is mechanistically plausible, given the conserved Keap1–Nrf2 axis, but direct evidence from oral tissues remains an area requiring further study [191,192,193,200]. Additional variability arises from differences in biofilm composition, biofilm maturity, host–cell models, and analytical endpoints, which may explain why some studies emphasize cytokine suppression, others antioxidant signaling, and others regenerative or antibiofilm effects as the dominant mechanism of action under specific experimental conditions.

Although curcumin-based nanoformulations have reported regenerative outcomes, such as increased collagen production and gingival fibroblast proliferation, long-term in vivo studies are needed to fully characterize the balance between regeneration and potential fibrotic responses in periodontal tissues. The available data indicate a regenerative trend through reduction in inflammatory mediators and osteoclast activity, but longitudinal studies in clinically relevant models are required to define the durability and safety of tissue remodeling [179,181,184].

The oral biofilm is a dynamic, multispecies ecosystem. Although curcumin-based nanoformulations appear promising in single-species models and in certain multispecies assemblies, translating these effects into the complex in vivo oral cavity remains challenging. Nanoformulations may enhance antibiofilm efficacy, but outcomes may be modulated by biofilm maturity, host factors, and the delivery device; therefore, carefully designed clinical studies are needed to establish efficacy in humans [175,180].

## 4. Antimicrobial Activity of Curcumin-Based Nanoformulations

Curcumin has attracted considerable interest because of its antimicrobial and antibiofilm properties in the context of oral health. When incorporated into nanoformulations, the solubility, stability, and bioavailability of curcumin are significantly improved, enabling enhanced interaction with targeted oral pathogens and deeper penetration into polymicrobial biofilms. The antimicrobial spectrum of curcumin-based nanoformulations commonly encompasses cariogenic bacteria, periodontopathogenic bacteria, and oral fungi such as *Candida albicans*, in agreement with the principal etiologic agents of dental caries, periodontitis, and oral candidiasis. In addition, nanoformulations may influence biofilm-related outcomes by reducing exopolysaccharide production, modulating the expression of adhesion-related genes, and enhancing carrier-mediated penetration into established biofilms. Accordingly, this chapter describes the main outcomes of curcumin-based nanoformulations and highlights (i) the antimicrobial spectrum relevant to oral health, including cariogenic bacteria such as *Streptococcus mutans*; periodontopathogenic bacteria such as *Porphyromonas gingivalis* and *Aggregatibacter actinomycetemcomitans*; and fungi such as *Candida albicans*; and (ii) the impact on biofilm formation, including reduction in exopolysaccharide production, modulation of the expression of adhesion-related genes, and improved biofilm penetration by nanocarriers. Building on the antimicrobial spectrum and antibiofilm actions of curcumin-based nanoformulations in the oral cavity, this chapter focuses on three interconnected aspects that influence translational potential: (1) nanoformulation synergism with other antimicrobials and the anticipated reduction in cytotoxicity through concentration-sparing effects; (2) comparative performance among nanoformulations, with emphasis on particle size, surface charge, and oral pH/stability; and (3) considerations regarding oral safety and cytotoxicity.

### 4.1. Antimicrobial Spectrum of Curcumin-Based Nanoformulations Against Major Oral Pathogens

A common feature of multiple curcumin-based nanoformulations is their ability to inhibit both planktonic and biofilm-associated forms of major oral pathogens, including *Streptococcus mutans*, *Porphyromonas gingivalis*, and *Candida albicans*. *Streptococcus mutans* and related cariogenic streptococci are key organisms in the development of dental caries, known for the synthesis of extracellular polysaccharides that underlie adhesive and robust biofilms on tooth surfaces. Curcumin-based nanoformulations have demonstrated activity against *S. mutans*, often accompanied by effects on biofilm-associated phenotypes such as glucosyltransferase-mediated EPS production and the expression of adhesion genes. For example, curcumin incorporated into solid nanoparticle-based systems demonstrated enhanced antimicrobial activity against *S. mutans*, with reduced viability and impaired biofilm formation compared with free curcumin, due to improved solubility and cellular uptake, thereby enabling more effective interaction with the bacterial cell envelope and intracellular targets [202]. In mono- and dual-species interactions modeling early caries and periodontal biofilms, curcumin exhibits inhibitory activity depending on the formulation and experimental conditions. For example, curcumin-loaded nanoformulations reduced the viability and biomass of *S. mutans* and *C. albicans* in mono- and dual-species biofilms, with the extracellular polymeric matrix (EPS) content and biofilm architecture being significantly altered, consistent with defective biofilm maturation [203]. In parallel, curcumin formulations have demonstrated activity against *P. gingivalis* (a key periodontal pathogen involved in adult periodontitis), inhibiting growth and disrupting biofilm at relatively low concentrations, thereby highlighting the potential to target periodontopathogenic communities [202,204,205,206].

One representative category of nanoformulations is that of curcumin-loaded polymeric nanoparticles, which have demonstrated improved solubility and targeted delivery to oral biofilms. Curcumin-loaded electrospun polymeric membranes inhibit *Pseudomonas aeruginosa* and *Streptococcus mutans* biofilms, confirming the efficacy of curcumin integrated into solid polymeric matrices for dental and medical device applications [207]. Studies using such systems report substantial antibiofilm effects against cariogenic and periodontopathogenic species, consistent with sustained antimicrobial activity in the complex oral environment [203,208]. Liposomal curcumin formulations similarly improve bioavailability and allow deeper penetration into cariogenic and periodontopathogenic biofilms, with observed reductions in the number of viable *S. mutans*, *P. gingivalis*, and *C. albicans* cells in mixed-species biofilms [203,209]. Curcumin-based solid lipid nanoparticles and nanoemulsions reduce biofilm biomass and EPS production, confirming improved diffusion through biofilm matrices due to nanoscale size and lipid-based carriers [203,210]. Moreover, chitosan-based curcumin nanocarriers exploit the intrinsic antimicrobial and bioadhesive properties of chitosan, facilitating adhesion to tooth surfaces and penetration into biofilms, with concomitant reductions in the viability of *S. mutans* and *C. albicans* in biofilms [211].

*Candida albicans*, the principal fungal agent in oral candidiasis, also falls within the antimicrobial scope of curcumin nanoformulations. Curcumin-based delivery systems have demonstrated antifungal activity against *C. albicans* and, in some studies, synergy with other antifungal constituents in nanoemulsions or nanoformulations, with reductions in viable microorganism counts and biofilm biomass in in vitro assays [202,203,212,213,214]. Concerning polymicrobial oral biofilms composed of *S. mutans*, *P. gingivalis*, and *C. albicans*, curcumin antibiofilm effects have been reported, including reductions in metabolic activity and biofilm mass in multispecies models and, in some cases, downregulation of virulence and adhesion-associated gene expression in interkingdom biofilms [206,212,215]. Integration of curcumin into nanostructured polymeric systems confers superior antibiofilm activity compared with the free compound. Curcumin-loaded chitosan-based nanoparticles inhibit polymicrobial biofilm formation and reduce the viability of microbial cells within the biofilm, demonstrating increased efficacy in *Candida albicans* and *Staphylococcus aureus* models [216,217].

Nanoformulations improve curcumin penetration into mature biofilms and enhance localized concentration at the biofilm–substrate interface. Curcumin-loaded nanoparticles or nanoemulsions exhibit superior diffusion through the extracellular polymeric substances of the biofilm and provide greater intracellular exposure in both bacterial and fungal cells, producing greater antibiofilm effects compared with free curcumin. In *S. mutans* biofilms, curcumin-based nanoformulations have demonstrated enhanced inhibition of biofilm formation and reductions in established biofilm mass through photodynamic activation or in combination with other antimicrobial modalities [202,206]. In multispecies interkingdom biofilms containing *C. albicans* and *S. mutans*, curcumin-based nanoformulations, particularly under photodynamic therapy (PDT) conditions, have demonstrated greater suppression of biofilm viability and matrix disruption, indicating that nanodelivery may potentiate the antibiofilm efficacy of curcumin against complex oral biofilms [212,215]. Graphene- and carbon-based nanocarriers, including curcumin-loaded graphene oxide compounds, have been highlighted as platforms that facilitate penetration into mature biofilms and may be functionalized to target key pathogens such as *P. gingivalis* and *A. actinomycetemcomitans*, as well as *C. albicans*, by reducing biofilm viability and disrupting EPS architecture in representative models [206,218].

Hybrid polymeric formulations, such as chitosan–TPP–curcumin nanoparticles, exhibit antibiofilm activity against both biofilm formation and mature biofilms, including those of resistant pathogens such as *Achromobacter* and *Burkholderia* [219]. In the field of oral applications, curcumin-loaded PLGA nanoparticles significantly improve antimicrobial activity and disruption of endodontic biofilm while maintaining a favorable biocompatibility profile [220]. These observations are complemented by data on PLGA–curcumin nanoparticles optimized for reducing bacterial adhesion and preventing early biofilms, highlighting the role of polymeric architecture in modulating microorganism–surface interactions [221]. Overall, these studies converge on the idea that nanoparticle-based polymeric formulations or coatings improve the local availability of curcumin and facilitate penetration into or interference with the extracellular polymeric substance (EPS) matrix of the biofilm, thereby resulting in clinically relevant antibiofilm activity.

Curcumin-loaded polymeric nanoparticles, liposomes, nanoemulsions, and Pluronic-based microcapsules represent a cross-section of curcumin nanoformulations with demonstrated antimicrobial effects against oral pathogens. Curcumin incorporated into Pluronic F-127 microcapsules demonstrated significant antimicrobial effects against *Streptococcus mutans* and *Candida albicans*, particularly under photodynamic conditions, with reported reductions in biofilm viability and biomass; similar delivery nanosystems have been associated with improved solubility and sustained release that prolong the duration of antimicrobial action against *S. mutans* and *C. albicans* [202]. In PDT-assisted approaches, curcumin acts as a photosensitizer, generating reactive oxygen species upon light activation, which leads to substantial reductions in the viability of *S. mutans* and *C. albicans* within biofilms; nanoencapsulation or nanoemulsion-based delivery may amplify these effects by increasing photosensitizer uptake and distribution within the biofilm matrix [222]. In multispecies biofilms, curcumin-based photosensitizers have been shown to downregulate virulence determinants in *C. albicans* and reduce adhesion in *S. mutans*, thereby weakening the interkingdom interactions that stabilize the biofilm [212,215].

For clarity, the main pathogen-specific antimicrobial and antibiofilm findings reported for curcumin-based nanoformulations are summarized in Table 2, including quantitative inhibitory parameters.

### 4.2. Inhibition of Biofilm Formation

A central mechanism by which curcumin-based nanoformulations disrupt biofilms is related to exopolysaccharide synthesis and adhesion. First, glucosyltransferases in *S. mutans* catalyze the production of extracellular polysaccharides that anchor cells to dental surfaces and promote the three-dimensional architecture of cariogenic biofilms. Curcumin, when delivered through nanoformulations, has been shown to downregulate the expression of adhesin-related genes and to attenuate the expression of genes involved in EPS production, thereby diminishing the structural integrity and adhesive capacity of the biofilm matrix. These observations are consistent with the finding that curcumin may downregulate glucosyltransferase activity (e.g., gtfB/C) and quorum-sensing-related gene networks, thereby contributing to reduced biofilm formation and persistence in *S. mutans*-containing systems [202,203,206,223]. Second, changes in the expression of genes involved in adhesion and surface attachment reflect the impact of curcumin on the initial stages of biofilm formation. In curcumin-treated biofilms, downregulation of adhesion-related gene transcription in *S. mutans* accompanies reduced adhesion to hydroxyapatite or tooth-mimicking surfaces, suggesting attenuation of the initial colonization stages [203,209]. In polymicrobial contexts, curcumin in nanodelivery platforms has demonstrated effects on interkingdom biofilms by altering the expression of virulence genes associated with *Candida albicans* adhesion and hyphal development, as well as bacterial adhesion determinants, suggesting a broad impact on the regulatory circuits governing early biofilm establishment and maturation in multispecies oral communities [203,212,213,215]. Third, improvements in biofilm penetration by nanocarriers are frequently reported for curcumin-loaded nanosystems, a feature attributed to nanoscale size, surface charge modifications (often cationic, as in the case of chitosan-based carriers), and bioadhesive polymers. Collectively, these properties facilitate deeper extrusion of curcumin into the biofilm matrix, enhancing antimicrobial exposure of sessile cells and EPS-rich regions. Empirical data show that curcumin-based nanoformulations produce more extensive biofilm penetration and greater reductions in biomass and EPS than native curcumin, especially in mixed-species biofilms representative of dental plaque communities [203,208].

Although the literature supports the role of curcumin-based nanoformulations in combating biofilms, some aspects warrant attention. Some studies report the antimicrobial effects of curcumin-based nanoformulations not only on planktonic bacteria but also on biofilms formed by cariogenic and periodontopathogenic species, with EPS reduction and downregulation of adhesion genes; however, the results may vary depending on biofilm age, species composition, formulation type, carrier material, curcumin loading, surface charge, medium composition, and the use of photodynamic activation. In certain multispecies settings, the antibiofilm effects of curcumin are strongest in the presence of light-activated PDT, whereas in the absence of PDT, activity may be reduced compared with monomicrobial systems. Notably, the potential synergy between curcumin and other phytochemicals or inorganic nanoparticles has also been highlighted, suggesting that combinatorial nanoformulations may exhibit superior antibiofilm efficacy, particularly in interkingdom biofilms involving *Candida* spp. and streptococci [203,212,215,222]. There is some heterogeneity regarding the reported impact on specific targets, such as EPS synthesis versus adhesion gene expression, reflecting differences in assay design (e.g., EPS quantification versus gene expression profiling) and the complexity of biofilm matrices. Nanoformulations improve curcumin bioavailability and its antifouling properties, thereby enhancing antimicrobial efficacy against the major oral pathogens mentioned above [202,206,212,223].

The demonstrated antimicrobial spectrum and biofilm-inhibitory actions of curcumin-based nanoformulations support their potential as adjuvant agents in oral care products intended for dental caries and periodontal disease. The ability to suppress EPS production and to downregulate adhesion gene expression translates into reduced biofilm initiation and maturation, potentially reducing cariogenic acid production and periodontal tissue destruction. Improved nanocarrier penetration into biofilms addresses a major limitation of conventional antimicrobials, namely the diffusion barrier represented by the extracellular matrix, thereby increasing the likelihood of eradicating sessile cells that drive recurrent infections [203,208,209]. In polymicrobial models, curcumin-based nanoformulations may disrupt interkingdom biofilm networks by targeting both bacterial and fungal adhesion and virulence pathways, a critical consideration for the clinical management of denture-associated stomatitis, periodontitis, and dental caries [202,212,215,222].

### 4.3. Synergy of Nanoformulations with Other Antimicrobials and Implications for Cytotoxicity

Curcumin delivered through nanoformulations may act synergistically with conventional antiseptics and antibiotics, thereby allowing reduction in the antimicrobial concentrations required and, consequently, in potential cytotoxicity. Curcumin delivery assisted by photodynamic therapy (curcumin-mediated photosensitization) demonstrates enhanced antimicrobial efficacy against oral biofilms compared with non-photosensitized formulations, owing to increased reactive oxygen species generation and improved biofilm penetration when curcumin is encapsulated or formulated as a nanocarrier [202,215,222]. Second, combination strategies associating curcumin with other phytochemicals or inorganic nanoparticles within a single nanodelivery platform have produced superior antibiofilm effects in interkingdom biofilms, suggesting that synergistic mechanisms may reduce the effective dose of each component and minimize cytotoxic exposure while maintaining antimicrobial performance [212,215,222]. Third, nanoformulations incorporating curcumin with metal-based nanoparticles (e.g., graphene oxide–curcumin composites or curcumin–silver carrier systems) have been proposed to facilitate improved biofilm diffusion and localized activity, which could allow lower curcumin loadings while achieving equal or greater efficacy against cariogenic and periodontopathogenic biofilms [206,218]. Overall, synergy may be achieved through (a) photodynamic activation, (b) integration with other antimicrobial agents within nanocarriers, and (c) the use of hybrid nanoplatforms that employ carrier-mediated transport to overcome EPS barriers, thereby allowing lower doses and a potentially reduced cytotoxic risk [202,206,212,215,218,222].

The magnitude of synergistic effects may depend on the specific formulation and the microbial consortium. In monomicrobial contexts, curcumin-loaded polymeric nanoparticles or liposomes often show improved activity relative to free curcumin, but the addition of an external photosensitizer or a co-antimicrobial may disproportionately amplify the effects in multispecies biofilms, where interkingdom interactions stabilize the biofilm matrix [202,215]. Moreover, although synergy offers a route to concentration reduction, some studies note formulation- and context-dependent variability in the degree of enhancement, underscoring the need for standardized comparative studies under clinically relevant conditions [202,206,212,222].

### 4.4. Comparison of Antimicrobial Activity Among Different Nanoformulations

Impact of particle size, surface charge, and stability at oral pH

Particle size influences diffusion through extracellular polymeric substances and penetration into the deeper layers of the biofilm. Within curcumin-based nanoformulations, smaller particles generally demonstrate superior diffusion and more uniform distribution within biofilms, correlating with stronger antibiofilm outcomes in *S. mutans* and interkingdom biofilms, particularly when combined with activation strategies such as photodynamic therapy. For example, nanoliposomes and nanoemulsions of approximately 100 nm have been associated with improved curcumin release profiles and enhanced antimicrobial performance compared with free curcumin or larger carriers, probably due to increased surface area and better penetration into EPS-rich matrices [176]. In addition, graphene- or carbon-based nanocarriers functionalized with curcumin have been highlighted for their ability to traverse mature biofilms and efficiently deliver curcumin to embedded cells, supporting the idea that nanoscale architecture modulates efficacy in complex biofilms [218].

Surface charge is a critical determinant of interactions with negatively charged microbial cell envelopes and the mucus-containing oral environment. Positively charged surfaces, such as chitosan-coated nanoparticles or polymers containing protonated amine groups, may favor electrostatic attraction to bacterial cell walls and mucosal surfaces, potentially improving contact time and uptake, thereby increasing antimicrobial activity against *Streptococcus mutans* and periodontal pathogens. This rationale is supported by nano-biointeraction studies and by reports indicating that cationic surfaces improve adhesion and retention in the oral cavity, which may translate into higher local curcumin concentrations and stronger antibiofilm effects [202,206,223]. However, excessively strong positive charge may increase the risk of cytotoxicity to host tissues, highlighting a formulation-specific balance between antimicrobial potency and biocompatibility. Although direct comparisons across studies are limited by heterogeneous designs, the convergent view is that positively charged biocompatible polymers, such as chitosan or Pluronic-based systems, may enhance antimicrobial performance while maintaining acceptable in vitro safety, provided that loading and release kinetics are optimized [202,222,223].

Stability at oral pH and in artificial saliva determines the practical performance of nanoformulations in the oral cavity. Curcumin solubility and chemical stability are significantly improved in nanoformulations, enabling sustained release and prolonged antimicrobial exposure under conditions resembling salivary pH (~6.2–7.4) and ionic composition. Studies reporting sustained release from nanoliposomes or polymeric nanoparticles indicate release profiles that support prolonged activity against *S. mutans* and *Candida* spp., consistent with improved antibiofilm outcomes in polymicrobial biofilms compared with free curcumin [176,202]. Stability considerations extend to formulation resistance in the presence of salivary enzymes and pH fluctuations; certain carrier systems (e.g., Pluronic-based microcapsules and liposomal formulations) have demonstrated robust performance across the tested pH ranges, whereas others may exhibit reduced stability under acidic conditions typical of cariogenic biofilms. These observations underscore the importance of selecting carriers with proven pH stability and simulated salivary longevity for clinically relevant efficacy [176,202,222].

Overall, when comparing nanoformulations, smaller particle size, positive surface charge, and demonstrable stability at oral pH emerge as favorable attributes for enhancing antimicrobial activity and biofilm disruption. However, improvements in one parameter do not necessarily translate into superior outcomes across all pathogen or biofilm models; formulation-specific performance must be evaluated in standardized multispecies models to establish the relative merits of different nanoformulations.

### 4.5. Oral Safety and Cytotoxicity Considerations

Safety and cytotoxicity considerations are essential for translating curcumin-based nanoformulations into oral care products. Curcumin is generally recognized for its favorable safety profile; however, challenges related to solubility and bioavailability have driven nanoformulation strategies aimed at reducing systemic exposure and enabling targeted local action within the oral cavity. Analyses of curcumin antimicrobial activity and toxicity indicate that, at antimicrobial-effective concentrations, cytotoxic effects on oral mucosal cells and fibroblasts may be minimized compared with conventional antimicrobials, especially when curcumin is delivered through nanoformulations designed for controlled release and reduced peak concentrations [225]. However, cytotoxicity is highly context-dependent, varying according to cell type, duration of exposure, carrier material, and loading efficiency, thus requiring explicit cytotoxicity testing for each formulation in relevant oral cell lines (e.g., keratinocytes, gingival fibroblasts) and in in vivo models prior to clinical application [225]. Moreover, the inclusion of metal-based nanoparticles (e.g., silver or zinc oxide) raises additional safety considerations because of potential cytotoxicity, systemic absorption, and concerns related to long-term exposure; consequently, safety assessments should comprehensively address cytotoxicity, genotoxicity, and inflammatory responses in oral tissues, including evaluations under salivary flow conditions and in reconstructed oral epithelium models [226,227,228,229]. Thus, a prudent, formulation-specific safety evaluation framework is supported, including in vitro cytotoxicity testing on relevant oral cell types, in vivo biocompatibility studies, and consideration of dosing strategies that minimize systemic exposure while maintaining antimicrobial efficacy [202,222,226,228].

Additional safety considerations refer to the potential for phototoxic effects within light-activated therapies and the need to verify that photodynamic regimens do not inadvertently damage host tissues. Evidence from studies on antimicrobial photodynamic therapy indicates that, when properly implemented, with appropriate light dosimetry and adequate photosensitizer concentrations, curcumin-based PDT may achieve substantial microbial reductions with selective targeting of biofilms; however, systematic evaluations of host tissue toxicity remain essential for clinical translation [222]. Another safety-related aspect concerns the long-term effects of repeated exposure to nanoformulations in the oral cavity, including alterations in the resident microbiota and mucosal tolerance, which require longitudinal in vivo investigations to ensure that beneficial commensals are preserved while pathogenic species are controlled [202,215].

Overall, the synergistic use of curcumin-based nanoformulations together with conventional antimicrobials and photodynamic strategies offers a viable pathway toward potent and targeted antimicrobial effects against key oral pathogens such as *Streptococcus mutans*, *Porphyromonas gingivalis*, and *Candida albicans*, while reducing the required concentrations and cytotoxic risk. Among nanoformulations, particle size, surface charge, and pH/stability emerge as critical determinants of antimicrobial performance, with smaller, positively charged carriers that remain stable in acidic/salivary environments providing superior biofilm penetration and sustained activity, although with formulation-dependent safety considerations. A general caution nevertheless remains: the results are heterogeneous with respect to formulations, experimental designs, and biofilm models. Consequently, standardized, direct comparisons in multispecies oral biofilm models, accompanied by rigorous cytotoxicity and in vivo evaluations, are essential for establishing clinically translatable curcumin-based nanoformulations for oral health applications.

## 5. Limitations, Challenges, and Future Research Directions

Although curcumin has attracted considerable interest because of its anti-inflammatory, antioxidant, antimicrobial, and anticancer properties, its clinical application has been hindered by fundamental pharmacokinetic and formulation-related challenges, particularly poor water solubility, chemical instability in physiological environments, rapid metabolism, and limited bioavailability. Over the past decade, nanoformulations have emerged as a central strategy for addressing these barriers; however, stability in the oral environment, mucosal retention, standardization and regulatory obstacles, clinical trial data, and the horizon offered by next-generation nanotechnologies remain critical and interconnected issues.

### 5.1. Stability of Nanoformulations in the Oral Environment

A major limitation affecting the translational potential of curcumin nanoformulations for oral health and other mucosal indications is the instability of curcumin under oral conditions and within the dynamic salivary environment. In the oral cavity, formulations are rapidly diluted by saliva, mechanical clearance, and exposure to enzymes and pH fluctuations, all of which may compromise drug integrity and release kinetics. Nanoformulations have been developed to protect curcumin from degradation, but the oral environment imposes unique stability requirements that differ from those associated with systemic administration. Nanoencapsulation improves the chemical and physical stability of curcumin by protecting it against hydrolytic and oxidative degradation and enabling controlled release; however, the salivary environment introduces an additional destabilizing paradigm because of dilution and protein interactions that may alter nanoparticle surfaces and drug release patterns. Protecting curcumin from enzymatic and environmental degradation is possible through various nanocarriers, such as polymeric nanoparticles, lipid-based systems, cyclodextrin complexes, and hydrogels, but the exact performance in the oral cavity, where salivary composition and flow may change rapidly, remains incompletely characterized and requires formulation-specific investigation across all formulation types [230,231,232,233,234,235,236,237,238,239,240,241]. Collectively, these studies suggest that salivary proteins may interact with nanoparticle surfaces, potentially altering aggregation state, mucoadhesion, or release, and that such interactions must be systematically characterized for clinically relevant oral doses and durations [239,242].

### 5.2. Bioavailability Issues and Mucosal Retention

Bioavailability and mucosal retention are closely linked in the oral and periodontal context, where short tissue contact time and rapid clearance limit therapeutic exposure. Native curcumin exhibits extremely low systemic bioavailability because of poor solubility, rapid metabolism, and extensive first-pass elimination; nanoformulations have consistently demonstrated substantial improvements in apparent bioavailability and tissue distribution in preclinical models and early clinical studies, which often translate into improved local retention at mucosal surfaces or enhanced systemic exposure after oral administration [232,236,237,239,241,242,243,244,245]. Despite these advances, many studies report that although relative bioavailability may be significantly increased (often by orders of magnitude), absolute plasma concentrations and tissue levels still depend on formulation composition, dosing regimen, and coadministration with absorption enhancers or metabolism inhibitors (e.g., piperine). In the context of periodontal applications, maintaining sustained mucosal contact remains challenging, and the need for mucoadhesive systems to prolong retention within periodontal pockets or gingival crevices has been repeatedly emphasized [224,231,232,233,236,237,239,242,243]. The use of mucoadhesive polymers, surface-modified nanoparticles, and localized delivery platforms (e.g., in situ gels or hydrogel matrices) is regarded as essential for transforming bioavailability gains into meaningful clinical outcomes in periodontal disease and associated oral mucosal disorders [136,244,246,247].

### 5.3. Standardization of Production and Regulatory Barriers

Another major challenge concerns the standardization and regulatory frameworks governing orally administered nanoformulations. The literature repeatedly mentions the lack of standardization across studies, including inconsistent reporting of critical physicochemical properties such as particle size, polydispersity index, drug loading, surface charge, and release profiles. This heterogeneity undermines reproducibility, comparability, and regulatory assessment, thereby complicating scale-up and quality control for clinical translation. Standardized processes are needed to align production methods, characterization protocols, and preclinical-to-clinical translation strategies to address regulatory ambiguity and facilitate clinical adoption.

The regulatory landscape for curcumin-based formulations is heterogeneous and depends strongly on product classification, intended use, and formulation claims. In the United States, curcumin products marketed as dietary supplements are regulated by the Food and Drug Administration (FDA) under the Dietary Supplement Health and Education Act (DSHEA), which does not require pre-market approval for safety and effectiveness in the same way as drug products. Oral-care products may also fall under cosmetic, drug, or combined cosmetic/drug regulation depending on their intended use and labeling claims. In the European Union, turmeric-containing products may, in some cases, follow the framework for traditional herbal medicinal products, while oral-care products without medicinal claims may instead fall under cosmetic legislation. For nanoformulations intended for therapeutic oral-health applications, these classification differences are particularly important because they determine the level of quality documentation, toxicological evaluation, labeling requirements, and clinical evidence needed for regulatory acceptance. Accordingly, successful translation of curcumin-based nanoformulations will require not only formulation optimization, but also early alignment with the appropriate regulatory pathway. The need for regulatory clarity is underscored by contexts involving translational barriers, including manufacturing scalability, reproducibility, and complex regulatory pathways for oral nano-products, all of which must ensure a balance among safety, efficacy, and product consistency [224,230,232,233,235,236,237,239,242,243,244]. In addition, regulatory agencies emphasize robust pharmacokinetic and toxicological data, standardized analytical methods for curcumin and its metabolites, and clear labeling of nanoformulation components, all of which impose additional burdens on developers [236,238,248]. Taken together, these considerations indicate that successful translation of curcumin-based nanoformulations into human oral applications will require harmonized formulation characterization, reproducible manufacturing workflows, robust pharmacokinetic and toxicological evaluation, and regulatory pathways capable of integrating product consistency with long-term safety and clinical efficacy.

From a commercialization perspective, market availability is currently more advanced for high-bioavailability curcumin products intended for general nutraceutical use than for oral-health-specific nanoformulations. Commercial examples include enhanced curcumin platforms such as Theracurmin^®^, NovaSOL^®^, Meriva^®^, and Longvida^®^, which are marketed primarily as supplements or formulation ingredients rather than as dental therapeutics. In parallel, turmeric-containing herbal medicinal products are recognized in some regulatory frameworks for traditional oral use, mainly for mild digestive complaints. By contrast, although curcumin-containing toothpastes, mouthwashes, and gels have been described and investigated for oral applications, the market for standardized nano-curcumin products specifically developed and broadly established for gingivitis, periodontitis, caries, or oral candidiasis remains limited. This gap further emphasizes the need for stronger clinical validation, formulation standardization, and regulatory alignment before broader commercialization in oral healthcare can be achieved [37,249,250,251,252,253,254].

### 5.4. The Need for Controlled Clinical Studies

An additional issue that should be considered when interpreting the current evidence base is the limited clinical predictability of many in vitro findings. Although in vitro studies are essential for identifying antimicrobial, antibiofilm, anti-inflammatory, and mechanistic effects of curcumin-based nanoformulations, they are typically performed under simplified conditions that do not fully reproduce the complexity of the oral environment. In particular, mono-species biofilms, short incubation periods, static exposure conditions, and the absence of salivary flow, host immune responses, tissue turnover, and patient-dependent behavioral variables may lead to an overestimation of therapeutic performance. As a result, reductions in microbial viability, exopolysaccharide production, or inflammatory signaling observed in vitro should be interpreted as supportive rather than definitive evidence of clinical efficacy. Translation into clinical benefit requires validation in multispecies models, ex vivo systems, animal studies, and ultimately well-controlled human studies.

Against this background, another critical gap is the lack of controlled clinical studies with adequate statistical power to evaluate curcumin nanoformulations under comparable, real-world conditions. Although several phase I/II studies and small-scale investigations have demonstrated safety, tolerability, and some signs of efficacy, particularly in metabolic disorders, cancer, and inflammatory conditions, the evidence base remains numerically limited and often heterogeneous in terms of study design. Future research must involve large, well-controlled cohort studies with standardized endpoints and direct comparisons with standard treatments or a placebo. Such studies should be stratified according to formulation type, dosage, and concomitant therapies in order to determine which nanoformulations provide clinically significant advantages, to establish optimal dosing, and to assess long-term safety and tolerability. In addition, direct comparisons with current standard-of-care regimens in periodontal therapy or systemic inflammatory conditions would be particularly informative for regulatory decision-making and clinical adoption [224,230,232,233,235,236,237,239,242,243,255,256].

### 5.5. Perspectives for Next-Generation Nanotechnologies

Looking ahead, smart and stimuli-responsive nanoplatforms are positioned to address several of the current limitations. Stimuli-responsive nanocarriers that react to local factors such as pH changes, enzymatic activity, or inflammatory microenvironments may enable on-demand release and formulation-specific enhancement of curcumin bioactivity, potentially improving both efficacy and safety. In the periodontal context, smart systems designed to release curcumin in response to the acidity or proteolytic activity of periodontal pockets could concentrate therapeutic action where inflammation and infection are greatest, thereby reducing systemic exposure and improving retention within periodontal pockets. Beyond single-agent carriers, combinatorial approaches, such as the co-delivery of curcumin with probiotics or other anti-inflammatory agents, are being explored in order to exploit synergistic mechanisms, combining the anti-inflammatory actions of curcumin with the microbiome-modulating effects of live biotherapeutics or prebiotics. Heterogeneous nanoassemblies that combine imaging capabilities with therapeutic delivery (theranostics) represent another avenue for improving patient stratification and treatment monitoring. Overall, next-generation strategies emphasize stimuli-responsive materials, combination therapies, and targeted delivery systems to achieve precise, durable, and clinically meaningful outcomes in periodontitis and associated mucosal diseases [136,224,231,232,233,235,236,237,239,242,243,244,256].

### 5.6. Future Directions and Convergent Opportunities

The horizon for curcumin nanoformulations includes several convergent opportunities. First, stimuli-responsive formulations promise site-specific release aligned with the inflammatory microenvironment or the acidic niches of periodontal disease, which could maximize local efficacy while minimizing systemic exposure. Second, combining curcumin with probiotics or other bioactive agents could exploit complementary mechanisms, namely microbiome modulation alongside anti-inflammatory action, to achieve more comprehensive management of periodontal disease and associated systemic inflammatory conditions. In parallel with carrier-based approaches, rational structural modification of curcumin through the development of synthetic analogues may represent a complementary strategy for overcoming the limitations of the native compound. Recent studies on mono-carbonyl curcumin analogues have shown that structural optimization may enhance biological activity and antioxidant performance in preclinical models, suggesting that future oral-health-oriented research may benefit from integrating analogue design with nanocarrier engineering [257,258,259,260].

Third, smart nanoplatforms incorporating imaging capabilities could enable real-time monitoring of drug localization and response, thereby informing personalized adjustments to therapy. Finally, standardization initiatives and regulatory harmonization will be essential for moving from preclinical promise to clinically approved products with clearly defined therapeutic roles in dentistry, periodontology, and mucosal medicine [136,224,231,232,233,235,236,237,239,242,243,244,245,256].

## 6. Conclusions

Curcumin-based nanoformulations represent a promising and rapidly expanding therapeutic strategy for oral health applications. By addressing the major limitations of native curcumin, including poor water solubility, chemical instability, rapid metabolism, and low bioavailability, nanocarrier systems substantially improve local delivery, mucosal retention, controlled release, and tissue exposure. Across the representative platforms reviewed herein, including polymeric nanoparticles, nanomicelles and nanoemulsions, solid lipid nanoparticles and nanostructured lipid carriers, nanogels, hydrogels, mucoadhesive films, and metallic or hybrid systems, nanoformulation consistently emerges as a key determinant of curcumin performance in the oral environment.

The available evidence indicates that these systems exert multitarget effects that are highly relevant to the pathogenesis of oral diseases. In addition to improving pharmacokinetic behavior, curcumin nanoformulations modulate major molecular pathways involved in periodontal and mucosal pathology, including suppression of NF-κB-driven inflammation, activation of antioxidant defenses through the Nrf2/HO-1 axis, attenuation of oxidative stress, and support of tissue repair and regenerative responses. Moreover, they demonstrate relevant antimicrobial and antibiofilm effects against major oral pathogens, including *Streptococcus mutans*, *Porphyromonas gingivalis*, *Aggregatibacter actinomycetemcomitans*, and *Candida albicans*, through reduced exopolysaccharide production, impaired adhesion, improved penetration into mature biofilms, and modulation of virulence-associated signaling. These combined properties position curcumin nanoformulations as attractive adjunctive candidates for the prevention and management of dental caries, gingivitis, periodontitis, oral candidiasis, and related mucosal inflammatory conditions.

Nevertheless, despite strong mechanistic and preclinical support, clinical translation remains limited by several unresolved challenges. These include insufficient standardization of formulation design and characterization, incomplete knowledge regarding stability in the dynamic oral environment, limited data on long-term mucosal safety and cytotoxicity, formulation-dependent variability in antimicrobial efficacy, and a lack of adequately powered controlled clinical studies. Future progress in this field will depend on the development of reproducible and regulatory-compatible manufacturing strategies, the optimization of mucoadhesive and stimuli-responsive delivery systems, and the integration of combinatorial approaches capable of enhancing both therapeutic precision and clinical durability. Overall, curcumin-based nanoformulations hold substantial potential to reshape current therapeutic strategies in dentistry, but their successful incorporation into clinical practice will require rigorous translational validation. From a pharmaceutical technology perspective, the comparative insights summarized herein may support the rational design and optimization of next-generation curcumin delivery platforms for oral applications.

## Figures and Tables

**Table 1:** Comparative advantages and limitations of the main types of curcumin-based nanoformulations.

Ref.	Nanoformulation Type	Advantages	Limitations
[51,53,129,131,138,143]	Polymeric nanoparticles	• Enhanced stability and protection: polymeric encapsulation may protect curcumin from hydrolytic/enzymatic degradation and may improve its chemical stability, thereby enabling more favorable pharmacokinetics and therapeutic performance. • Sustained and controlled release with targeting potential: polymeric matrices allow modulation of release kinetics and, through surface modification, active targeting (e.g., ligand-assisted uptake). • Improved cellular uptake and bioactivity compared with free curcumin: in vitro studies have demonstrated superior activity in cancer models compared with free curcumin, consistent with improved transport and intracellular retention. The anticancer and anti-inflammatory potential of polymeric curcumin systems and related nanocarriers has also been emphasized. • Technological maturity and scalability: polymeric systems have demonstrated proof of concept for scale-up, including scalable bottom–up processes for PLGA-based curcumin nanoparticles, as well as documented efforts related to production scale-up and stability assessment, thereby supporting translational potential and collectively illustrating the feasibility of moving from laboratory-scale to pilot-scale production.	• Complexity and reproducibility: polymer synthesis, surface functionalization, and batch-to-batch variability may complicate production consistency and regulatory approval. • Data on antimicrobial activity: while nanocurcumin and related polymeric systems often show improved anticancer activity and general therapeutic potential, the antimicrobial performance of curcumin-loaded polymeric nanoparticles has been reported less consistently; thus, their antimicrobial advantages remain less well defined relative to their gains in solubility and bioavailability. • Cost and scalability: although explicit evidence exists for the scalable production of polymeric nanoparticles (particularly PLGA-based systems), total manufacturing costs and industrial-scale processes depend on polymer selection, payload, and surface chemistry; scale-up remains carrier- and process-specific.
[51,129,142,143]	Nanocells and nanoemulsions	• Superior solubility and rapid action: nanomicelles and nanoemulsions are highly effective in solubilizing curcumin in aqueous media, thereby facilitating improved oral and parenteral bioavailability as well as rapid tissue absorption. • Relative simplicity and formulation versatility: these soft self-assembled systems can be prepared by relatively simple methods and may be tailored in terms of release and stability, contributing to broader translational potential. • Permeability and absorption benefits: by improving drug solubility and enabling intimate contact with mucosal/epithelial surfaces, these carriers may enhance permeability and systemic exposure compared with native curcumin.	• Challenges related to physical stability and dilution: nanomicelles, in particular, may undergo dilution-induced disassembly and loss of active substance (the critical micelle concentration phenomenon), which may compromise in vivo performance and shelf life under certain conditions. • Scalability and long-term stability: although scalable, micellar systems and nanoemulsions require robust stabilization strategies to preserve integrity during storage and administration. • Data on antimicrobial efficacy: comparative data regarding the specific antimicrobial performance of nanomicellar/nanoemulsion curcumin formulations are not reported uniformly; antimicrobial potential remains an area of ongoing research, with broader discussions focusing on the therapeutic scope of curcumin in nanoformulations.
[53,130,132,133,134,136,138,139,143,144,145]	Solid–lipid nanoparticles and nanostructured lipid carriers (SLN/NLC)	• Improved stability and protection through the lipid matrix: SLNs protect curcumin from degradation and enable controlled/prolonged release while maintaining biocompatibility. • Improved bioavailability and loading efficiency: lipophilic curcumin benefits from the lipid matrix, resulting in improved oral absorption and systemic exposure compared with free curcumin. • Scalability and manufacturability: SLN-based formulations have high potential for scalable manufacturing using heat- or solvent-assisted processes and relatively simple lipid excipients, thereby supporting industrial translation. • The NLC concept for addressing SLN limitations: nanostructured lipid carriers are designed to overcome crystallinity-related drug expulsion and to provide higher drug loading and more flexible release, thereby addressing some of the limitations observed with purely crystalline SLNs.	• Effects of crystallinity and polymorphism: the crystalline state of lipids in SLNs may influence drug loading and release; DSC and polymorphic transitions are commonly analyzed to optimize performance, highlighting a key aspect of SLN stability that may affect reproducibility and release kinetics. • Potential for limited drug loading compared with NLCs: although SLNs provide protection and controlled release, their drug-loading capacity may be more limited than that of certain NLC formulations; the NLC approach explicitly aims to expand loading capacity and tailor release behavior. • Scale-up considerations: although scalable, lipid-based production still requires careful control of lipid crystallinity, phase behavior, and process parameters to ensure consistent particle size and drug loading across batches. • Data on antimicrobial efficacy: the antimicrobial performance of curcumin-loaded SLNs/NLCs is not reported uniformly; the emphasis remains on solubility, bioavailability, and anticancer potential, while antimicrobial outcomes require additional comparative data.
[51,129,137,141]	Nanogels, hydrogels, and mucoadhesive films	• High loading capacity, tunable release, and mucosal targeting: nanogels and hydrogels provide versatile matrices for curcumin encapsulation with controlled release; mucoadhesive films enable prolonged residence time at mucosal surfaces and facilitate noninvasive administration routes (e.g., buccal, ocular, vaginal). • Sustained and stimuli-responsive release: several studies demonstrate sustained release from dextrin-based nanogels and gelatin-based nanogels, with release profiles determined by network properties and, in some cases, by external stimuli (e.g., magnetic guidance, photothermal triggers in gelatin-based systems). • Biocompatibility and potential for localized delivery: hydrogel/nanogel platforms frequently use biocompatible polymers and naturally derived components, supporting their potential translational use for localized cancer therapy or mucosal delivery. • Production and scalability: hydrogels and nanogels are compatible with scalable formulation strategies in many cases, and microfluidic or controlled-assembly approaches are being explored to improve reproducibility and manufacturing feasibility.	• Diffusion and release control: gel networks may impose diffusion barriers, potentially complicating the achievement of rapid therapeutic exposure when desired; release kinetics must be carefully tailored to match the intended application. • Mechanical and swelling behavior: hydrogels and nanogels are sensitive to swelling, ionic strength, and pH, all of which may affect stability and in vivo performance. • Maturity and translational gaps: although nanogels/hydrogels are well established in research, their translation into commercial products often lags behind lipid and polymeric nanoparticle systems because of manufacturing, sterilization, and regulatory considerations.
[129,135,136,143]	Metal or hybrid nanosystems	• Multimodal capabilities and targeted delivery: inorganic or hybrid nanosystems (e.g., mesoporous silica nanoparticles, magnetically guided cores, or metal oxide/hybrid composites) provide opportunities for multimodal therapy, imaging, and externally guided targeting; examples include curcumin-loaded mesoporous silica nanoparticles for targeted delivery and improved stability, as well as magnetic core systems for magnetically guided uptake into cancer cells. • Antimicrobial and imaging potential: inorganic and hybrid systems may confer antimicrobial activity or enable imaging modalities that complement the pharmacology of curcumin. • Mature concepts in specific niches: some metallic/hybrid systems have advanced to preclinical demonstrations of targeted delivery or combination therapy (e.g., magnetic nanoparticles with a silk fibroin core and shell) and exhibit advantages related to sustained release and localization in cell cultures or animal models. • Production and scalability considerations: inorganic/metallic systems often require more complex and specialized synthesis and purification steps, which may translate into higher production costs and more stringent qualification requirements for industrial-scale manufacturing.	• Biocompatibility, safety, and regulatory obstacles: inorganic materials may raise long-term concerns regarding biocompatibility or clearance, and regulatory pathways for inorganic/hybrid nanocarriers are often more demanding than those for polymeric or lipid-based systems. • Stability and reproducibility: the synthesis of inorganic nanostructures with uniform size, shape, and surface chemistry may be technically challenging, affecting batch-to-batch consistency and scale-up. • Antimicrobial efficacy and niche applications: although metallic/hybrid systems may exhibit antimicrobial effects or enable imaging, robust direct antimicrobial comparisons among nanoformulations are rare; antimicrobial performance tends to be context-dependent and application-specific.

**Table 2:** Summary of representative studies reporting antimicrobial and antibiofilm effects of curcumin-based nanoformulations (oral- and non-oral-pathogens).

Nanoformulation Type	Target Pathogen(s)	Experimental Model	Main Antimicrobial/Antibiofilm Effect	Quantitative Parameter (MIC/IC_50_/MBIC or Equivalent)	Ref.
Curcumin-containing mouthwash nanoemulsion	*Streptococcus mutans*	In vitro saliva-conditioned pellicle biofilm model, with *S. mutans* inocula of 10^4^ and 10^6^ CFU/mL and treatment for 24 h or 48 h	Inhibited *S. mutans* biofilm formation in a concentration-dependent manner; the 10% formulation showed the best activity, but overall efficacy was lower and declined after 48 h	MIC of biofilm formation = 10% *v*/*v*; biofilm inhibition at 24 h: ~39% (5%) and 45% (10%) at 10^4^ CFU/mL, and ~24% (5%) and 35% (10%) at 10^6^ CFU/mL	[82]
Curcumin-loaded bacterial cellulose/alginate/gelatin composite films (BCAGG-C1 to BCAGG-C4)	*Escherichia coli*, *Staphylococcus aureus*	In vitro antibacterial surface assay with 48 h incubation and agar plate colony counting	Curcumin-loaded films showed concentration-dependent antibacterial activity against both pathogens; BCAGG-C3 had activity comparable to commercial Bactigras^®^ after 48 h; greater inhibition against *S. aureus* than *E. coli*	Viable bacterial counts after 48 h (log CFU/mL): *E. coli*: ~2.1 (BCAGG-C1), ~1.4 (BCAGG-C2), ~1.1 (BCAGG-C3), ~0.6 (Bactigras^®^); *S. aureus*: ~0.8 (BCAGG-C1), ~0.1 (BCAGG-C2), ~0.1 (BCAGG-C3), ~0.1 (Bactigras^®^)	[110]
Combination of silver nanoparticles and curcumin nanoparticles (Cur-SNPs)	*Pseudomonas aeruginosa*, *Staphylococcus aureus*	In vitro biofilm formation inhibition and preformed-biofilm eradication assays, and CFU analysis	Cur-SNPs showed stronger antibiofilm activity than Cur-NPs or AgNPs alone, both in preventing biofilm formation and in removing established biofilms	Established biofilms: 50% disruption at 100 μg/mL Cur-SNPs, while 500 μg/mL Cur-NPs alone was needed for the same effect; Biofilm formation: *S. aureus*: 85% inhibition at 20 μg/mL Cur + 2.5 μg/mL Ag and 100% inhibition at 30 μg/mL Cur + 3.75 μg/mL Ag; *P. aeruginosa*: complete inhibition at 40 μg/mL Cur + 5 μg/mL Ag; Mature biofilm biomass: ~70% reduction for both pathogens at 400 μg/mL Cur + 50 μg/mL Ag; *P. aeruginosa* CFU decreased from 9.5 × 10^8^ to 2.5 × 10^3^ CFU/cm^2^ after Cur-SNP treatment	[117]
Silver-decorated curcumin-loaded polymeric micelles (PM-Ag-Cur)	*Pseudomonas aeruginosa*, *Staphylococcus aureus*	In vitro OD600 bacterial viability assay after 12 h, plus PI membrane-damage staining after 4 h	PM-Ag-Cur showed the strongest concentration-dependent antibacterial activity against both pathogens, exceeding PM-Ag and PM-Cur; membrane-damaging bacterial killing confirmed	Bacterial viability at 500 μg/mL ~18% for *P. aeruginosa* and ~27–28% for *S. aureus* with PM-Ag-Cur	[118]
Curcumin-conjugated silica nanoparticles (curc-NPs)	*Pseudomonas putida*	In vitro 24 h planktonic growth assay plus developing and mature biofilm assays	Showed little antibacterial activity against planktonic cells but significantly enhanced antibiofilm activity vs. free curcumin, reducing both developing and mature biofilms and lowering sessile-cell viability	Biofilm formation was inhibited by up to 50%, and mature biofilms were disrupted by up to 54% at 2.5 mg/mL; sessile-cell viability reduced to ~55% of control in developing biofilms	[123]
Curcumin-loaded self-microemulsifying drug delivery system (Curcumin-E1E_SMEDDS5e)	*Escherichia coli*, *Staphylococcus aureus*	In vitro antibacterial testing	The optimized curcumin-loaded SMEDDS showed antibacterial activity against *E. coli* and *S. aureus*; no antibiofilm assay reported	MIC = 48.62 μg/mL (*E. coli*) and 97.65 μg/mL (*S. aureus*)	[142]
Nanoliposomal curcumin	*Streptococcus mutans*, polymicrobial salivary biofilm	In vitro MIC/MBC testing and AAA biofilm model with single-species and polymicrobial oral biofilms	Nanoliposomal curcumin showed stronger antibacterial and antibiofilm activity than free curcumin, with lower MIC/MBC values and greater reduction in CFU counts in both single-species and polymicrobial biofilms	*S. mutans*—MIC 250 μg/mL (free curcumin) vs. 222 μg/mL (nanoliposomal curcumin); MBC 2 mg/mL vs. 1 mg/mL; in polymicrobial biofilm, nanoliposomal curcumin at 0.500 mg/mL caused ~1-log CFU reduction compared with control; single-species biofilm CFU reduction was also greatest at 0.500 mg/mL	[176]
Nano-curcumin (nanomicellar curcumin formulation; SinaCurcumin^®^)	*Streptococcus mutans*	In vitro planktonic and preformed dentin-slab biofilm model; CFU counting after treatment with nano-curcumin ± blue LED-mediated PDT	Nano-curcumin-mediated PDT significantly reduced *S. mutans* viability, less effective than free curcumin- or erythrosine-mediated PDT; antimicrobial activity was greater in planktonic than biofilm cultures	Nano-curcumin 3 g/L—40.0% reduction (planktonic) and 16.2% reduction (biofilm) without light; 82.3% reduction (planktonic) and 54.9% reduction (biofilm) with PDT	[186]
Curcumin–sophorolipid nanocomplex (CU-SL)	*Candida albicans*	In vitro planktonic susceptibility, pre- and post-adhesion biofilm inhibition assays	Sub-MIC CU-SL significantly inhibited fungal adhesion, biofilm development/maturation, and filamentation; free curcumin showed no biofilm inhibition at sub-MIC concentrations	9.37 µg/mL (sub-MIC) significantly inhibited adhesion and biofilm development, reduced attached cells/mm^2^ (*p* < 0.05), and abolished filamentation	[187]
Curcumin-loaded nanoformulation	*S. mutans* + *Candida albicans*	Mono- and dual-species biofilms	Reduced viability and biomass; altered EPS content and biofilm architecture; downregulation of virulence-related genes	MBEC_50_ = 0.5 mM	[203]
Curcumin-loaded electrospun polymeric membrane	*Pseudomonas aeruginosa*, *S. mutans*	Biofilm formation assay on electrospun membranes	Inhibited biofilm formation without noticeable inhibition of planktonic bacterial growth	Biofilm inhibition: 38 ± 3% (*P. aeruginosa*) and 47 ± 3% (*S. mutans*)	[207]
Curcumin-loaded chitosan nanoparticles (CSNP-Cur)	*Candida albicans*, *Staphylococcus aureus*, Polymicrobial biofilm of *C. albicans* + *S. aureus*	In vitro MIC assay, biofilm formation inhibition, mature biofilm disruption, CFU assay, CLSM/SEM on silicone	Antibacterial, antifungal, and antibiofilm activity; almost complete inhibition of biofilm formation at 200 μg/mL; stronger disruption of preformed mono- and polymicrobial biofilms than free curcumin	MIC: 200 μg/mL (free curcumin) and 400 μg/mL (CSNP-Cur) for both *S. aureus* and *C. albicans*; preformed biofilm reduction at 400 μg/mL: 88.48% (*S. aureus*), 91.38% (*C. albicans*), 84.36% (polymicrobial biofilm); CFU reduction in polymicrobial biofilm: 83.56% (*S. aureus*), 86.54% (*C. albicans*)	[217]
Curcumin–chitosan nanocomplexes with sodium tripolyphosphate in magnetic nanoparticles (Cur-Chi-TPP-MNP)	*Achromobacter xylosoxidans*, *Burkholderia cepacia complex*, *Stenotrophomonas maltophilia*	Biofilm inhibition and eradication assays, tested in combination with trimethoprim–sulfamethoxazole (TMP-SXT)	Inhibited biofilm formation in all three non-fermenting Gram-negative pathogens and showed lower activity against established biofilms	Biofilm inhibition: 37.5 µg/mL (*A. xylosoxidans*), 18.75 µg/mL (*B. cepacia complex*), 4.69–18.75 µg/mL (*S. maltophilia*); Biofilm eradication—150–300 µg/mL for all three strains	[219]
PLGA-loaded curcumin polymeric nanoparticles (NP + Cur)	*Enterococcus faecalis* *Streptococcus oralis* *Actinomyces viscosus*	In vitro MIC/MBC testing and antibiofilm evaluation in single- and multispecies preformed endodontic biofilms	Photoactivated curcumin-loaded PLGA nanoparticles showed antibacterial activity and significantly reduced the viability of preformed single- and multispecies endodontic biofilms	325 μg/mL of photoactivated NP + Cur produced the greatest reduction in bacterial viability	[220]
Curcumin-encapsulated PLGA nanoparticles (nanoprecipitated NPC-5 and electrosprayed EPC-5)	*Streptococcus mutans*	In vitro viable cell count assay with CFU/mL, reduction percentage, and log reduction after 6 h and 24 h	Both nanoparticles showed strong antibacterial and antibiofilm activities; and reduced biofilm biomass/thickness	Efficacy against *S. mutans*: 99.9% reduction (3-log reduction) at 24 h for both nanoparticles	[221]
Metal–curcumin complexes (Cu-CUR, Zn-CUR, Fe-CUR)	*Pseudomonas aeruginosa*	In vitro MIC, cell-growth, and biofilm-formation assays, with anti-quorum-sensing/virulence evaluation	Greater antimicrobial activity than free curcumin and significantly inhibited *P. aeruginosa* biofilm formation; Cu-CUR was the most active complex	MIC = 62.5 µg/mL for metal–curcumin complexes vs. 125 µg/mL for free curcumin; biofilm inhibition = 45–90% overall; Cu-CUR at 1/4 MIC reduced biofilm formation by 90%; growth at 1× MIC was reduced to 1.5–3.3% depending on the complex	[223]
Nanocurcumin-coated gutta-percha cones	*Escherichia coli*	In vitro agar diffusion antibacterial assay	Nanocurcumin-coated gutta-percha showed greater antibacterial activity than curcumin-coated gutta-percha, while uncoated gutta-percha showed no antibacterial effect	Mean inhibition-zone values: 0.45 (uncoated gutta-percha), 0.97 (curcumin-coated gutta-percha), 1.55 (nanocurcumin-coated gutta-percha); F = 6056, *p* < 0.00001	[224]

## Data Availability

No new data were created or analyzed in this study. Data sharing does not apply to this article.

## Abbreviations

The following abbreviations are used in this manuscript:

WHO	World Health Organization
CHX	Chlorhexidine
COX-2	Cyclooxygenase 2
NAFLD	non-alcoholic fatty liver disease
iNOS	inducible nitric oxide
PLGA	poly(lactic-co-glycolic) acid
PCL	poly-ε-caprolactone
PLA	polylactic acid
PEG	polyethylene glycol
EPS	extracellular polymeric substances (matrices)
SLN	solid–lipid nanoparticles
NLC	nanostructured lipid carriers
ROS	reactive oxygen species
AgNPs	silver nanoparticles
CuNPs	copper nanoparticles
ADN	deoxyribonucleic acid
DSC	differential scanning calorimetry
PDT	photodynamic therapy
LPS	lipopolysaccharide
